# Specification and Diversification of Pericytes and Smooth Muscle Cells from Mesenchymoangioblasts

**DOI:** 10.1016/j.celrep.2017.05.019

**Published:** 2017-05-30

**Authors:** Akhilesh Kumar, Saritha Sandra D’Souza, Oleg V. Moskvin, Huishi Toh, Bowen Wang, Jue Zhang, Scott Swanson, Lian-Wang Guo, James A. Thomson, Igor I. Slukvin

**Affiliations:** 1 Wisconsin National Primate Research Center, University of Wisconsin-Madison, Madison, WI 53715, USA; 2 Great Lakes Bioenergy Research Center, University of Wisconsin-Madison, Madison, WI 53703, USA; 3 Neuroscience Research Institute, University of California, Santa Barbara, Santa Barbara, CA 93106, USA; 4 Department of Surgery, University of Wisconsin-Madison, Madison, WI 53792, USA; 5 Morgridge Institute for Research, Madison, WI 53707, USA; 6 Department of Cell and Regenerative Biology, School of Medicine and Public Health, University of Wisconsin-Madison, Madison, WI 53707, USA; 7 Department of Molecular, Cellular & Developmental Biology, University of California, Santa Barbara, Santa Barbara, CA 93106, USA; 8 Department of Pathology and Laboratory Medicine, University of Wisconsin-Madison, Madison, WI 53792, USA; 9 Lead Contact

## Abstract

Elucidating the pathways that lead to vasculogenic cells, and being able to identify their progenitors and lineage-restricted cells, is critical to the establishment of human pluripotent stem cell (hPSC) models for vascular diseases and development of vascular therapies. Here, we find that mesoderm-derived pericytes (PCs) and smooth muscle cells (SMCs) originate from a clonal mesenchymal progenitor mesenchymoangioblast (MB). In clonogenic cultures, MBs differentiate into primitive PDGFRβ^+^ CD271^+^CD73^−^ mesenchymal progenitors, which give rise to proliferative PCs, SMCs, and mesenchymal stem/stromal cells. MB-derived PCs can be further specified to CD274^+^ capillary and DLK1^+^ arteriolar PCs with a proinflammatory and contractile phenotype, respectively. SMC maturation was induced using a MEK inhibitor. Establishing the vasculogenic lineage tree, along with identification of stage- and lineage-specific markers, provides a platform for interrogating the molecular mechanisms that regulate vasculogenic cell specification and diversification and manufacturing well-defined mural cell populations for vascular engineering and cellular therapies from hPSCs.

## In Brief

Kumar et al. find that mesodermal pericytes and smooth muscle cells in human pluripotent stem cell cultures originate from a common endothelial and mesenchymal cell precursor, the mesenchymoangioblast. They show how different lineages of mural cells are specified from mesenchymoangioblasts and define stage- and lineage-specific markers for vasculogenic cells.

## Graphical Abstract

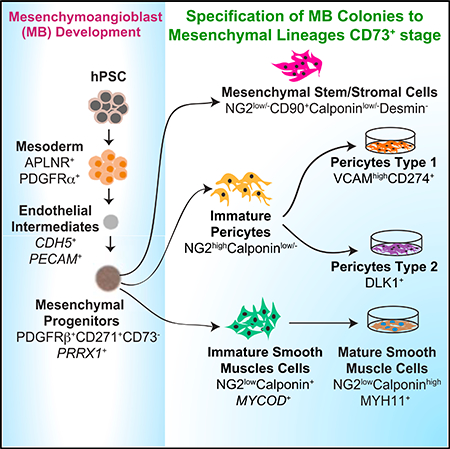

## INTRODUCTION

During embryonic development, the first vascular network, the capillary plexus, is formed in the yolk sac by endothelial cell precursors derived from nascent mesoderm (Risau and Flamme, 1995). Later, the development of mature blood vessels involves a complex process of vascular remodeling that depends on the proliferation and sprouting of new vessels from preexisting ones, and recruitment of mural cells, pericytes (PCs), and vascular smooth muscle cells (SMCs), in an autocrine-paracrine manner (Rossant and Howard, 2002). PCs reside within microvessels, whereas SMCs contribute to the vascular wall of larger vessels. Although all endothelial cells, with the exception of corneal, are derived from mesoderm (Noden, 1978, 1990), SMCs and PCs have much more diverse origins that include mesoderm and neural crest as two major sources (Armulik et al., 2011; Majesky et al., 2011). Recent advances in human pluripotent stem cell (hPSC) technologies made it possible to generate all types of vascular cells (endothelial, PCs, and SMCs) ex vivo to study vascular biology and diseases (Bajpai et al., 2012; Cheung et al., 2012; Dar et al., 2012; Levenberg et al., 2002; Orlova et al., 2014; Patsch et al., 2015; Prasain et al., 2014). However, understanding vasculogenic cell development in hPSC cultures and applying hPSC-based progenitor cell therapies to the vascular wall are hampered by the lack of knowledge about the hierarchy of vasculogenic progenitors and markers that can be used to discriminate PCs, SMCs, mesenchymal stem/stromal cells (MSCs) and their direct ancestors. In our prior studies, we demonstrated that the onset of mesenchymo- and vasculogenesis from hPSCs (human embryonic stem cells [hESCs] and human induced pluripotent stem cells [hiPSCs]) is defined by the emergence of the clonal precursor mesenchymoangioblast (MB), which originates from APLNR^+^PDGFRα^+^ primitive posterior mesoderm (Vodyanik et al., 2010). MBs are identified by their capacity to form fibroblast growth factor 2 (FGF2)-dependent compact colonies of mesenchymal/mesodermal cells in a semisolid medium, which are capable of differentiating into endothelial cells and MSCs with chondro-, osteo-, and adipogenic differentiation potentials (Vodyanik et al., 2010). Here, we report that, in addition to endothelial and skeletogenic differentiation potentials, MBs have the capacity to differentiate into SMCs and PCs. Based on these studies, we identified a lineage tree of mesodermal progenitors, which can be applied to explore the molecular pathways leading to specification and diversification of mesenchymal lineage cells in humans.

## RESULTS

### Induction and Specification of PCs and SMCs from MBs

In our prior studies (Vodyanik et al., 2010), we revealed that APLNR^+^PDGFRα^+^ primitive posterior mesoderm induced from hPSCs in coculture with OP9 stromal cells acquires the potential to form FGF2-dependent compact spheroid colonies in semisolid medium with a MSC and endothelial potentials that define MBs. MB colonies are formed through VE-cadherin^+^ endothelial intermediates (Figure S1A) that morph into colonies composed of CD146^+^CD271^+^CD73^−^ mesodermal progenitors with a transcriptional profile resembling posterior/lateral plate mesoderm-derived embryonic mesenchyme (Vodyanik et al., 2010). When transferred to adherent serum-free cultures and cultured with FGF2, MB colonies gave rise to CD73^+^CD105^+^CD31^−^CD45^−^ MSC lines (Vodyanik et al., 2010).

**Figure 1.**
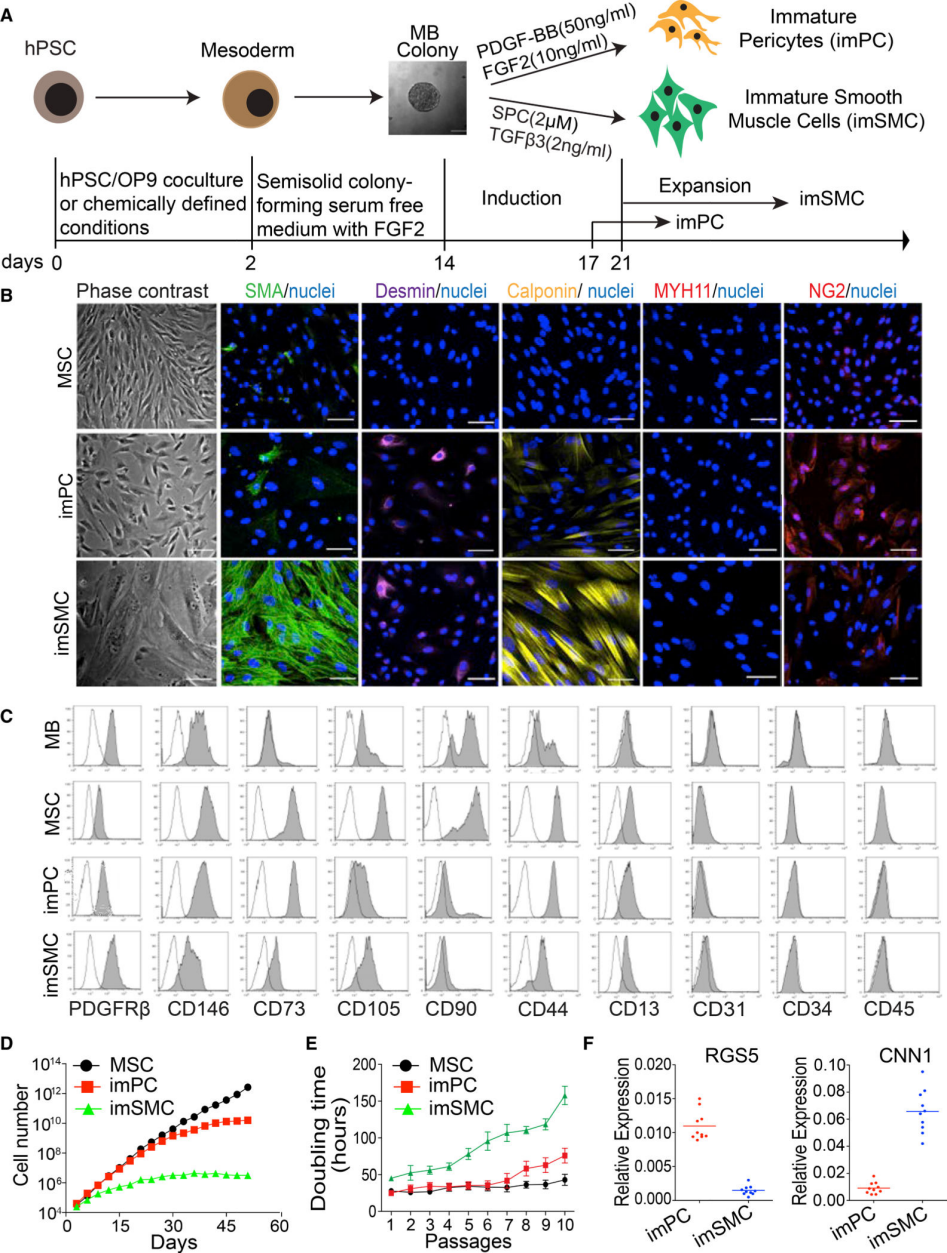
Characterization of imPCs and imSMCs Generated from hPSCs through the MB Pathway (A) Schematic diagram of the differentiation protocol used to generate imPCs and imSMCs from hPSCs. Following mesoderm induction, hPSCs were transferred into semisolid medium with FGF2 to induce formation of MB colonies. MB colonies were collected on day 12 of clonogenic culture and plated on fibronectin and collagen-coated plastic in the presence of the indicated factors to induce imSMCs and imPCs. After reaching a monolayer, imPC and imSMCs were passaged in Pericyte and EGM-2 medium, respectively. Photograph shows MB colony (scale bar, 50 μm). (B) Immunohistochemistry analysis of SMC and PC markers in MSCs, imPCs, and imSMCs derived from H1 hESCs. Nuclei (blue) were co-stained with DAPI. Scale bar, 50 μm. MSCs were generated from MB colonies by culture in serum-free medium with FGF2 (Vodyanik et al., 2010). (C) Flow cytometry analysis of MB-derived mesenchymal colonies (MB), MSCs, imPCs, and SMCs. (D) Expansion potential of MB-derived MSCs, imPCs, and imSMCs; representative experiment is shown. (E) Doubling time of MB-derived mesenchymal cells. Results are mean ± SE of three independent experiments. (F) qRT-PCR analysis of *RGS5* and *CNN1* expression in imPCs and imSMCs obtained from individual MB colonies. Each dot on the graph represents values by imPCs or imSMCs obtained from an individual MB colony. Horizontal lines show average expression levels for all tested colonies (n = 10). Images in (B) and histograms in (C) are representative of ten experiments. See also Figures S1–S3.

Because embryonic mesenchyme originating from lateral plate/splanchnic mesoderm contributes to the formation of PCs and SMCs (reviewed in Armulik et al. [2011] and Majesky [2007]), we hypothesize that MBs have the potential to differentiate into mural cells. To test whether MBs have PC potential, we collected MB colonies generated from H1 hESCs differentiated on OP9 and cultured them in the presence of platelet-derived growth factor (PDGF)-BB (Figure 1A), because PDGF-B/PDGFRβ signaling plays the most critical role in PC development in vivo (Levéen et al., 1994; Soriano, 1994). Indeed, in these conditions, MB colonies produced cells strongly expressing PC markers NG2, PDGFRβ, CD13, and CD146, and negative/weakly expressing smooth muscle actin (SMA) and calponin (Figures 1B and 1C), thereby confirming that these cells acquired the PC phenotype. The expression of CD73, CD13, and NG2 markers distinguished PCs from their mesenchymal precursors within MB colonies, whereas high NG2 and low CD90 and CD105 expression distinguished PCs from MSCs (Figures 1B, 1C, S1B, and S1C). PCs generated from MBs can be cultured for up to 12 passages with gradual senescence observed during 8–12 passages (Figures 1D and 1E). Because these cells were proliferative, we designated them as immature PCs (imPCs). Using the described approach and the OP9 coculture system, we were able to induce similar cells from H9-EGFP hESCs and fibroblast-derived DF-19–9-7T iPSCs (Figures S2A and S2B). Recently, we developed chemically defined conditions for hematoendothelial differentiation that reproduced the distinct steps of hematovascular development observed in the OP9 system (Uenishi et al., 2014). We found that MB colonies generated from H1 hESCs and blood-derived IISH2i-BM9 iPSCs following mesoderm induction in defined conditions also possessed the potential to differentiate into NG2^high^SMA^low^Calponin^low/−^MYH11^−^ imPCs (Figures S2C and S2D). Typically, we were able to produce up to 1.04 × 10^12^ imPCs from 10^6^ hPSCs using our MB-based differentiation protocols.

To test the SMC potential of MB colonies, we transferred them into media supplemented with known inducers of SMC differentiation, transforming growth factor β3 (TGFβ3) and sphingosylphosphorylcholine (SPC) (Chambers et al., 2003; Cheung et al., 2012) (Figure 1A). As shown in Figure 1B, the cells in these cultures became much larger as compared to imPC cultures, acquired typical rhomboid morphology of synthetic SMCs, and upregulated the expression of typical SMC molecules SMA and calponin. In contrast to imPCs, SMCs exhibited lower expression of NG2 (Figure 1B), CD146, CD13, CD73, and CD44 (Figures 1C and S1C). As the generated SMCs were proliferative and expandable for up to seven passages (Figures 1D and 1E), we designated these cells as immature SMCs (imSMCs). The capacity of MBs to produce SMCs was consistent among different hPSC lines including H9-EGFP hESCs and fibroblastderived DF-19–9-7T iPSCs (Figures S2A and S2B). The imSMCs were also obtained from MB colonies generated from H1 hESCs and blood-derived IISH2i-BM9 iPSCs, following mesoderm induction in serum- and feeder-free defined conditions (Uenishi et al., 2014) (Figures S2C and S2D). Typically, we were able to generate up to 1.8 × 10^9^ imSMC from 10^6^ hPSCs using our MB-based protocols.

To demonstrate the origin of mural cells from clonal MB precursors, we isolated individual MB colonies and cultured them in PC or SMC conditions and then performed qRT-PCR analysis for *RGS5* PC and *CNN1* SMC markers. As shown in Figure 1F, 10 out of 10 individual MB colonies produced PCs (high *RGS5*/low *CNN1*) in PC conditions, and 10 out of 10 individual MB colonies produced SMCs (low *RGS5*/high *CNN1*) in SMC conditions, indicating that each MB colony has the potential to differentiate into both PCs and SMCs. To estimate the frequency of progenitors within MB colonies, we collected individual MB colonies and performed limiting dilution assay in PC, SMC, and MSC conditions. These studies revealed that the readout frequency of PC progenitors was 1 per 4.5 cells, of SMC progenitors was 1 per 8 cells, and of MSC progenitors was 1 per 2.8 cell (Figure S3), thereby suggesting that MB colonies contain multipotential mesenchymal progenitors.

### Maturation and Specification of PCs from MB-Derived imPCs

**Figure 2.**
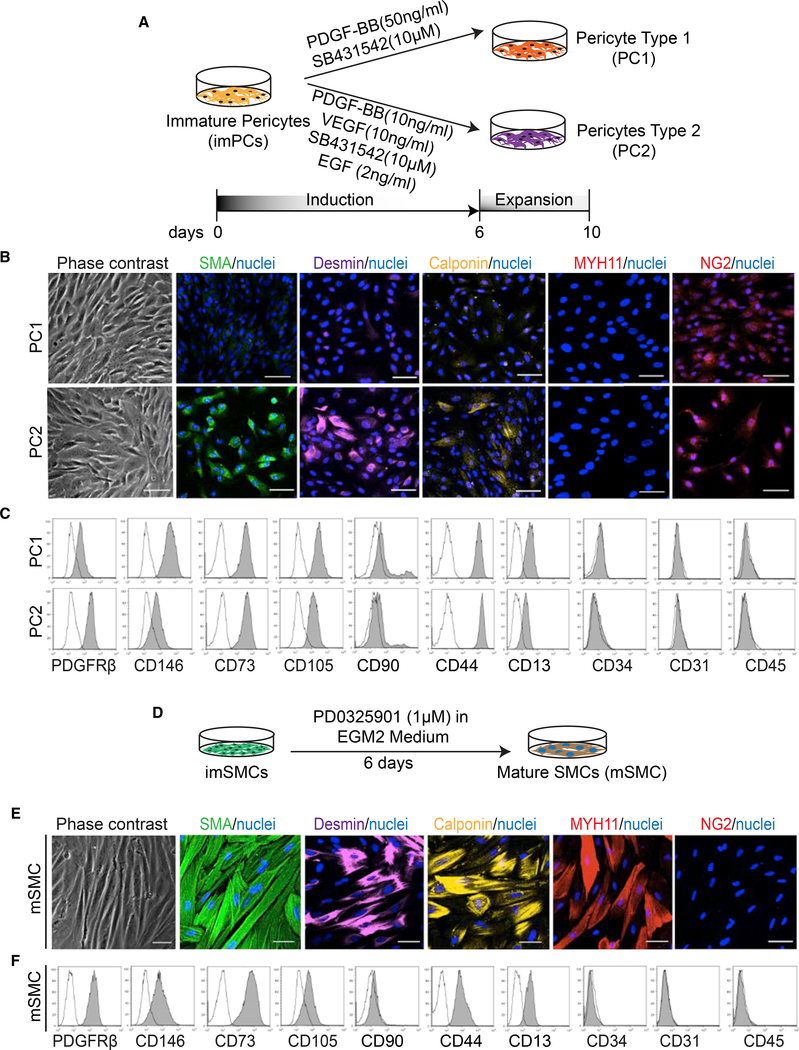
Generation and Characterization of PC1, PC2, and mSMCs (A) Schematic illustration of the strategy used for induction of PC1 and PC2 from imPCs. (B) Immunohistochemistry analysis of SMC and PC markers in PC1 and PC2. Nuclei (blue) were co-stained with DAPI. Scale bar, 50 μm. (C) Flow cytometry analysis of PC1 and PC2. (D) Schematic illustration of the strategy used for the induction of SMC maturation. (E) Immunohistochemistry analysis of SMC and PC markers in mSMCs. Nuclei (blue) were co-stained with DAPI. Scale bar, 50 μm. (F) Flow cytometry analysis of mSMCs. Images in (B) and (E) and histograms in (C) and (F) are representative of three experiments.

In the human body, PCs are phenotypically and functionally heterogenous, with the cells of small arterial, venous, and capillary vessels in addition to tissue-specific vascular beds, exhibiting distinct features. In situ phenotypic analysis demonstrated that capillary PCs are NG2^+^αSMA^−^, venular NG2^−^αSMA^+^, and arteriolar NG2^+^αSMA^+^ (Crisan et al., 2008, 2009). Some NG2^+^Calponin^−^ PCs express chemoattractants, nuclear factor κB (NF-κB), and inflammatory cytokines (Stark et al., 2013). Because PC recruitment to vessels and their maturation status is regulated by PDGF, TGFβ, epidermal growth factor (EGF), and vascular endothelial growth factor (VEGF) signaling (reviewed in Armulik et al. [2011]), we explored whether modulators of these pathways can affect specification of imPCs (Figure 2A). We found that treatment of imPCs with PDGF-BB and TGFβ-signaling inhibitor SB431542, induced PCs with NG2^+^SMA^low/−^Desmin^low/−^Calponin^low/−^MYH11^−^ capillary phenotype, which we designated PC type 1 (PC1). In contrast, treatment of imPCs with SB431542, PDGF-BB, VEGF, and EGF, induced PCs with NG2^high^SMA^+^Desmin^+^Calponin^low/−^MYH11^−^ arteriolar phenotype, which we designated PC type 2 (PC2) (Figure 2B). Both types of PCs expressed typical PC markers and lacked the expression of hematoendothelial markers (Figure 2C). We therefore concluded that imPCs could be specified to different types of PCs.

### Induction of Mature SMCs from MB-Derived imSMCs

As described above, imSMCs generated from MBs had the typical features of synthetic SMCs, including rhomboid morphology, high proliferative potential, and non-uniform expression of SMC proteins (Owens et al., 2004). Because an active ERK cascade is essential for the support of protein synthesis in SMCs and their growth (Servant et al., 1996), we hypothesized that inhibition of MEK could promote imSMC maturation. Thus, we treated imSMCs with the MEK inhibitor PD0325901 (Figure 2D). After treatment, imSMCs lost their proliferative potential, acquired a more elongated morphology and MYH11 expression, and substantially upregulated expression of other SMC markers (Figures 2E and 2F). Morphologic evaluation revealed well-organized contractile proteins spanning the SMC body, thereby confirming that MEK inhibitor induces efficient maturation of SMCs.

### Molecular Profiling of MBs and Their Vasculogenic Progeny Revealed a Unique Molecular Signature

**Figure 3.**
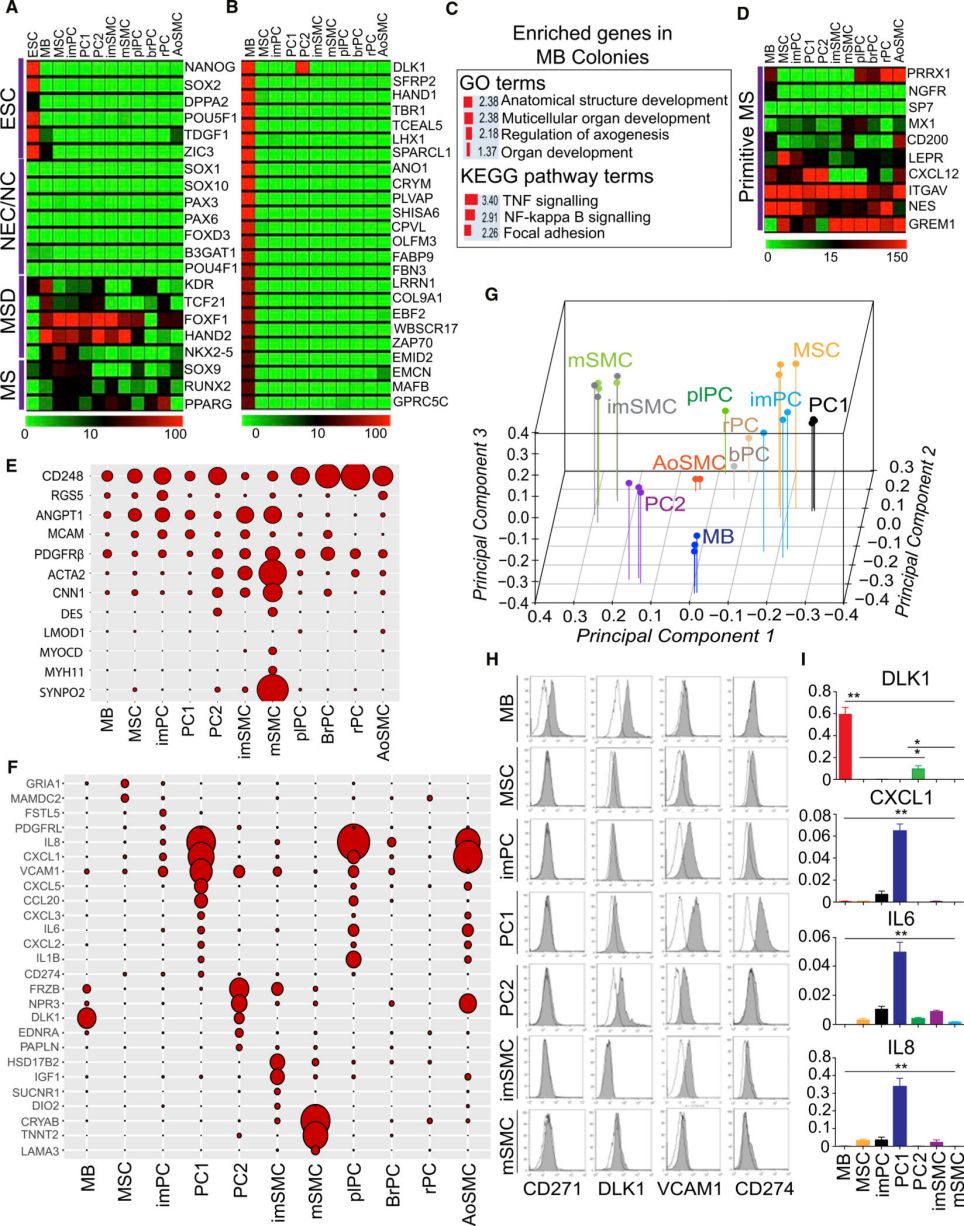
Gene Expression Profiling Reveals a Unique Molecular Signature of MB-Derived Mural Cells (A) Heatmap of a selected set of genes associated with the development of germ layers and their derivatives. MS, Mesenchyme; MSD, mesoderm; NEC, neuroectoderm; NC, neural crest. Gene expression is estimated in transcripts per million (tpm) values. (B) Heatmaps for the 24 representative genes uniquely overexpressed in MB colonies (MB) as compared to MB-derived mesenchymal cells (MSCs, PCs, and SMCs). A pool of 18 libraries representing triplicates of PC1, PC2, imPC, imSMC, mSMC, and MSC was used as a reference to detect MB-specific expression markers. (C) The classification of genes uniquely overexpressed in MB colonies as compared with downstream progeny. Enrichment scores (expressed as −log_10_(FDR)) for Gene Ontology (GO) and KEGG pathways are shown. (D) Heatmaps show the expression of genes associated with the most primitive mesenchymal cells (MS). (E) Balloon graph shows relative expression of typical PC and SMC genes in hPSC-derived mesenchymal cells. The largest area corresponds to 610.75 units. ACTA2 was downscaled four times for more representative visualization of other markers. (F) Balloon graph shows relative expression of selected genes differentially expressed in MB-derived MSCs and mural cells. The largest area corresponds to 2, 275 units. (G) PCA of transcriptome data for the 11 cell types (points are colored according to individual sample label). (H) Flow-cytometric analysis confirms the differences in the expression of the indicated molecules found by RNA-seq analysis. (I) qRT-PCR confirmed the differences in gene expression found by RNA-seq. Results shown are mean ± SE of three independent experiments (**p < 0.001, *p < 0.01). plPC, placental PC; brPC, brain PC; rPC, retinal PC; aoSMC, aortic SMC. Heatmaps in (A), (B), (C), and balloon graphs in (E) and (F) depict mean of three independent experiments with hPSC-derived mural cells, two independent experiments with aortic SMCs, and RNA-seq data of somatic PCs from a single experiment. See also Figures S4–S6.

To identify stage-specific differentiation markers and confirm the identity of differentiated cells, we performed RNA-sequencing (RNA-seq) analysis on the hPSC-derived mural cells and somatic PCs from brain, placenta, and retina. Transcriptional profiling revealed that all differentiated progeny from hPSCs lacked expression of pluripotency genes (Figure 3A). Consistent with prior findings (Vodyanik et al., 2010), MB colonies expressed *KDR*, *TSF21*, *FOXF1*, *HAND2*, and *NKX-2.5* genes typically found in posterior/lateral plate mesoderm and lacked the expression of genes associated with neuroectoderm development (Figure 3A). The most distinct feature of MB colonies as compared to downstream progeny was very high expression of noncanonical Notch ligand gene *DLK1*, which is involved in tissue morphogenesis, and twist family bHLH gene *HAND1*, which is critical for the development of the heart and extraembryonic mesoderm (Figures 3B and 3F; Data S1). Other genes uniquely expressed in MB colonies included *TBR1* and *LHX* transcription factors that are involved in regulation of many developmental processes, and extracellular matrix *EMCN* (Figure 3B). Genes overexpressed in MB colonies were enriched in anatomical structure development, multicellular organ development, and regulation of axogenesis Gene Ontology (GO) categories, and tumor necrosis factor (TNF), NF-κB signaling, and focal adhesion Kyoto Encyclopedia of Genes and Genomes (KEGG) signaling pathways (Figure 3C). Next, we analyzed whether MB colonies expressed genes known to be present in mouse and human primitive bone marrow mesenchymal progenitors, including *Prrx1* (Greenbaum et al., 2013), *Nes* (Méndez-Ferrer et al., 2010), *Lepr* (Ding et al., 2012; Zhou et al., 2014), *Cxcl*12 (Ding and Morrison, 2013), *Sp7* (Liu et al., 2013), *Mx1* (Park et al., 2012), *Grem1* (Worthley et al., 2015), *CD200* and *Itgav* (Chan et al., 2015), *NGFR* (Cattoretti et al., 1993; Tormin et al., 2011), and *MCAM* or *CD146* (Sacchetti et al., 2007). We found that expression of *PRRX1* and *NFGR* uniquely distinguished cells within MB colonies from differentiated progeny (Figure 3D), whereas *MCAM* (*CD146*) gene and protein were broadly expressed throughout all stages of differentiation (Figures 1C, 2C, 2F, 3D, and 3E). MB colonies were essentially lacking expression of typical SMC genes, including *MYOCD*, *MYH11*, *CNN1*, and *SYNPO2* (Figure 3E), and expressed lower levels of *RGS5*, *CD248*, *RGS5*, *ANGPT1*, and *ACTA2* (SMA) as compared to their progeny (Figure 3E). Using immunofluorescent analysis, we confirmed no expression of SMA, Calponin, NG2, and MYH11 SMC and PC markers in cells composing MB colonies at the protein level (Figure S1B). Based on functional analysis of the differentiation potential of MB colonies (MSCs [Vodyanik et al., 2010], PCs and SMCs [current studies]), and their unique gene expression profile signified by expression of *HAND1*, *TBR1*, and *LHX* morphogenesis regulators and primitive mesenchymal markers *NGFR*, *PRRX1*, and low/lack of expression of specific SMC and PC markers, we concluded that mesenchymal cells within MB colonies represent a distinct stage of mesenchymogenesis capable of specification into a broad range of vasculogenic and skeletogenic cells.

Following MB differentiation into imPCs, we observed an increase in expression of 1,182 genes, including typical PC genes *RGS5*, *MCAM*, and *CD248* with no or minimal upregulation of SMC genes (Figure 3E; Data S2). KEGG pathway assignment of these upregulated genes demonstrated enrichment in focal adhesion, extracellular matrix (ECM)-receptor interaction, p53 signaling, citrate cycle, axon guidance, and cell adhesion molecules (Figure S4A). MB differentiation into imSMCs was associated with upregulation of 3,070 genes, including *ACTA2* and *CNN1* SMC genes (Figure 3E; Data S2), enriched in focal adhesion, ECM-receptor interaction, TGFβ, PI3K-Akt, and HIF-1-signaling pathways KEGG categories (Figure S4B). As compared to imSMCs, imPCs upregulated 2,485 genes (Data S2), which were enriched in KEGG pathways involving Ras, Rap1, and FoxO signaling pathways, cell adhesion, and axon guidance categories (Figure S4C). Genes found to be more highly expressed in imSMCs as compared to imPCs, were shown to be enriched in PI3K-Akt, and TGFβ signaling pathways, complement and coagulation cascade, and aldosterone-regulated sodium reabsorption KEGG categories (Figure S4C). Overall, the observed differences in the gene expression profiles were consistent with PC versus SMC designation of MB-derived mural cells. To identify subset-specific markers, we have developed a hyperbolic-exponential filtering procedure that utilizes a sliding fold change threshold, which is dependent on expression level, thereby allowing for selection of the maximal number of meaningful genes, avoiding a bias toward low-expressed genes. Using this procedure, we found that imSMCs uniquely expressed a high level of *IGF1* (insulin growth factor 1), *HSD17B2* (hydroxysteroid 17-beta dehydrogenase 2), and type 2 iodothyronine deiodinase (*DIO2*) gene, which is found in human coronary artery and aortic SMCs (Mizuma et al., 2001) (Figure 3F). In contrast, imPCs demonstrated unique expression of follistatin like 5 (*FSTL5*) and platelet-derived growth factor receptor-like (*PDGFRL*) genes, whereas expression of *GRIA1* and *MAMDC2* genes distinguished MSCs from imPCs and imSMCs (see Data S3 for the complete list of uniquely expressed genes). The total numbers of uniquely overexpressed genes in individual subset and commonalities in overexpressed genes between subsets visualized using matrix layout for all intersections of six mesenchymal cell subsets are presented in Figure S5A.

Comparative transcriptome analysis of PC1 and PC2 revealed distinct differences in the expression of genes associated with inflammation and SMCs (Figures 3E and 3F). Whereas PC1 downregulated the expression of SMC genes, they significantly upregulated the expression of chemoattractants, including *CXCL1*, *CXCL5*, and *IL8* (*CXCL8*), programmed cell death 1 ligand *PDL1* (*CD274*), inflammatory cytokines, including *IL1B* and *IL6*, and adhesion molecule *VCAM1*. KEGG assignment of the genes overexpressed in PC1 revealed enrichment of pathways related to inflammation, including cytokine-cytokine receptor interaction, chemokine, TNF, and NF-κB signaling pathways (Figure S4D). This “proinflammatory” gene expression profile has been found characteristic of NG2^+^Calponin^−^ PCs (Stark et al., 2013). In contrast, PC2 expressed higher levels of *ACTA2*, *DES*, and *CNN1* SMC genes but was lacking expression of more specific *MYOCD* and *MYH11* SMC genes (Figure 3E). In addition, PC2 distinctly expressed *DLK1* found in fetal arteriolar PCs (Khan et al., 2016), Wnt-binding protein *FRZB*, natriuretic peptide receptor *NPR3*, extracellular matrix protein papilin (*PAPLN*), and endothelin receptor type A (*EDNRA*) gene, which is involved in regulation of long-lasting vasoconstriction (Figure 3F). KEGG pathway analysis of the genes overrepresented in PC2 versus PC1 revealed enrichment in vascular SMC contraction, TGFβ signaling, purine metabolism, and axon guidance categories (Figure S4D). Thus, the PC2 gene expression profile was more consistent with contractile arteriolar PCs.

Transition from an imSMC to mature SMC (mSMC) stage was associated with a significant upregulation of the typical SMC genes, including *MYH11*, *MYOCD*, *MYLK*, *MYH11*, *LMOD1*, *SYNPO2*, *TAGLN*, *CNN1*, and *ACTA2* (Figures 3E and S5B), and genes enriched in the KEGG categories ECM-receptor interaction, PI3K-Akt signaling pathway, focal adhesion and vascular SMC contraction, consistent with SMC maturation (Figure S4E). Interestingly, maturation of SMCs was associated with the high expression of cardiac-specific gene *TNNT2* (Figure S4E), which is also expressed in aorta during embryonic development (Jin et al., 2011). The observed differences in the expression of *CNN1*, *ACTA2*, *MYH11*, *MYOCD*, *TAGLN*, *RGS5*, *PDGFRB*, and *ANGPT1* SMC and PC genes between mural cell populations generated from H1 hESCs was confirmed using qPCR (Figure S5C). In addition, qPCR analysis revealed a similar pattern of SMC and PC gene expression in mural cell populations obtained from H9 hESCs and blood-derived IISH2i-BM9 iPSCs (Figure S5C).

To determine the relationship between in vitro generated cells and their in vivo counterparts, we performed principal-component analysis (PCA) of the transcriptomes of somatic PCs, versus the cells differentiated from hESCs. As shown in Figures 3G and S6A, SMCs and imPCs and PC1 were positioned more distal to MB colonies and clustered on opposite sides of the PCA plot, consistent with the observed diversification of SMCs and PCs from MBs. Contractile PC2 was positioned closer to MB colonies between SMCs and non-contractile PC1. hESC-derived PC1 were positioned closer to somatic PCs but farther from somatic aortic SMCs.

Based on comparative analysis of gene expression in MB colonies and MB-derived mesenchymal cells (Figures 3E and 3F and S5A; Data S3) we selected genes that could be used as identifying markers for MB-derived mural cell subsets, including *CD271*, *VCAM1*, and *CD274*, and analyzed their expression by flow cytometry. Consistent with their gene expression profiles, MB colonies could be discriminated from other cell subsets by the expression of CD271, whereas high expression of CD274 and VCAM1 distinguished PC1, and the expression of DLK1 along with the lack of CD271 distinguished PC2 (Figure 3H). The differences in *DLK1*, *CXCL1*, *IL6*, and *IL8* expression observed by RNA-seq in H1 hESC-derived mural cells were confirmed using qRT-PCR (Figure 3I). To ensure the reproducibility of these findings, we also demonstrated that similar markers could be successfully applied to discriminate mural cell subsets generated from H9 hESCs and blood-derived IISH2i-BM9 iPSCs (Figures S6B–S6E).

### Functional Characterization of MB-Derived Mural Cells In Vitro

**Figure 4.**
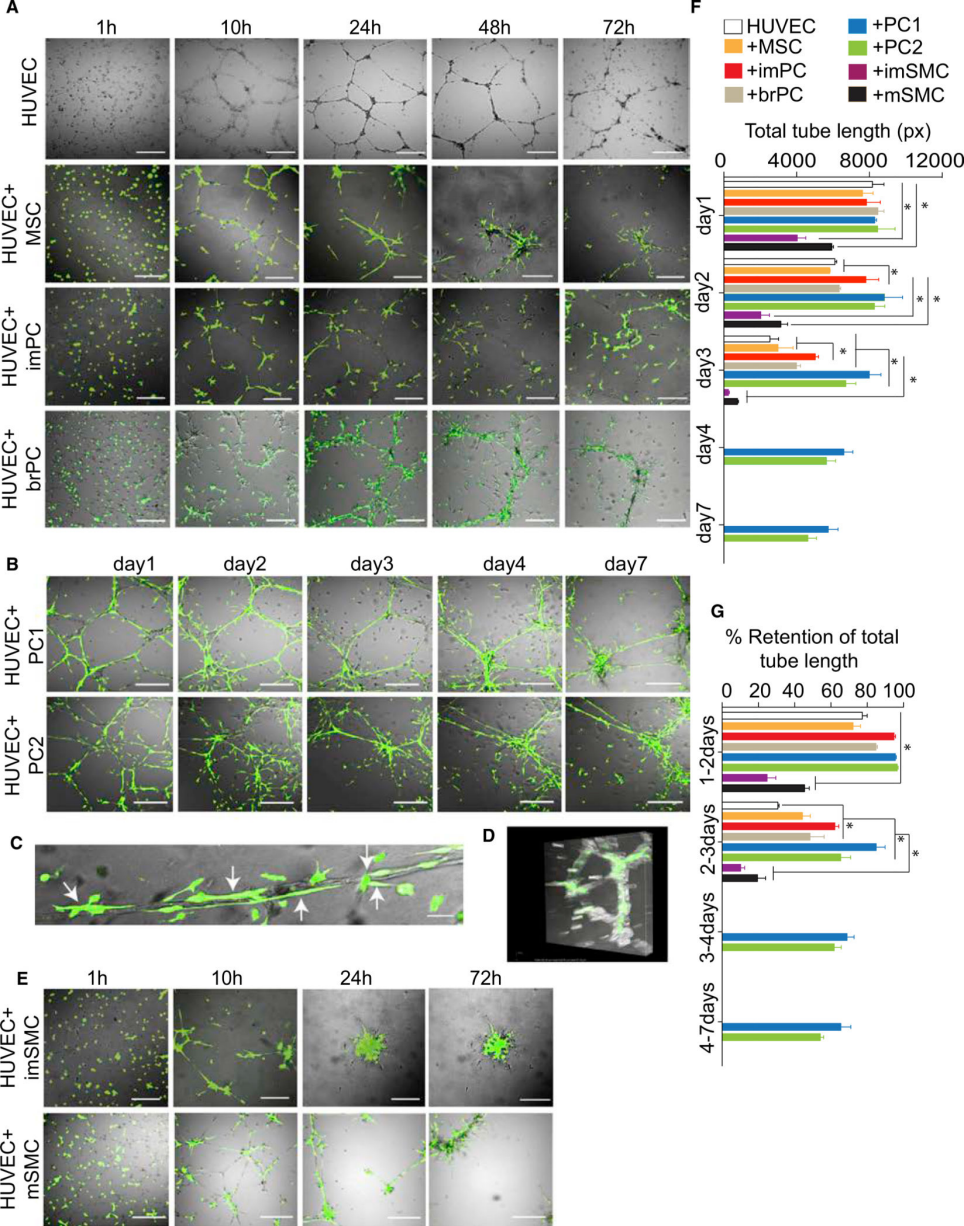
Vascular Tube-Stabilizing Potential of MB-Derived Mural Cells (A) HUVECs were cultured alone or cocultured with H9-EGFP hESC-derived MSCs, imPCs, or PKH67-labeled brain PCs (brPC) in pre-solidified Matrigel in EGM2 media. The cells were then photographed at the indicated time points using fluorescent microscope. Scale bar, 100 μm. (B) HUVECs were cocultured with H9-EGFP hESC-derived PC1 and PC2 photographed using fluorescent microscope at the indicated time points. Scale bar,100 μm. (C) Close-up view depicting tubular structures formed by HUVECs that are closely associated with PC1. Arrows point to PCs co-aligned with endothelial tubes. Scale bar, 10 μm. (D) 3D volumetric image of tubules formed by HUVECs in presence of PC1 (see also Movie S2). (E) HUVECs were cocultured with H9-EGFP hESC-derived imSMCs and mSMCs photographed using fluorescent microscope at the indicated time points. Scale bar, 100 μm. (F and G) Quantification of cumulative tube length (F) and retention of cumulative tube length (G) at indicated time intervals. Results are mean ± SE of three independent studies (*p < 0.01).

To study the functional properties of MB-derived mural cells, we evaluated their potential to stabilize vascular tubes in vitro. As shown in Figure 4A, tubes formed by human umbilical vein endothelial cells (HUVECs) in Matrigel were unstable and mostly dissolved after 72 hr. Although all in vitro-generated cells aligned along tubes when added to HUVECs, they had different effects on tube stability. The imPCs supported the tubes during 72 hr of culture, whereas MSCs had little effect on tube stability (Figure 4A; Movie S1). The tube-supporting capacity of imPCs was comparable to that of somatic brain PCs. Interestingly, the PC1 and PC2 demonstrated more profound effect on tube stability and supported tubes for up to 7 days (Figure 4B). As illustrated in Figure 4C at high magnification and with 3D imaging (Figure 4D; Movie S2), PCs align along and wrap around the luminized vascular tube structures, mimicking the position of PCs in vessels in vivo. In contrast, SMCs pulled tubes apart following alignment (Movie S3), thereby leading to rapid regression of the tubular network (Figure 4E). These observations were confirmed by the evaluation of the total tube length and its retention capacity (Figures 4F and 4G).

**Figure 5.**
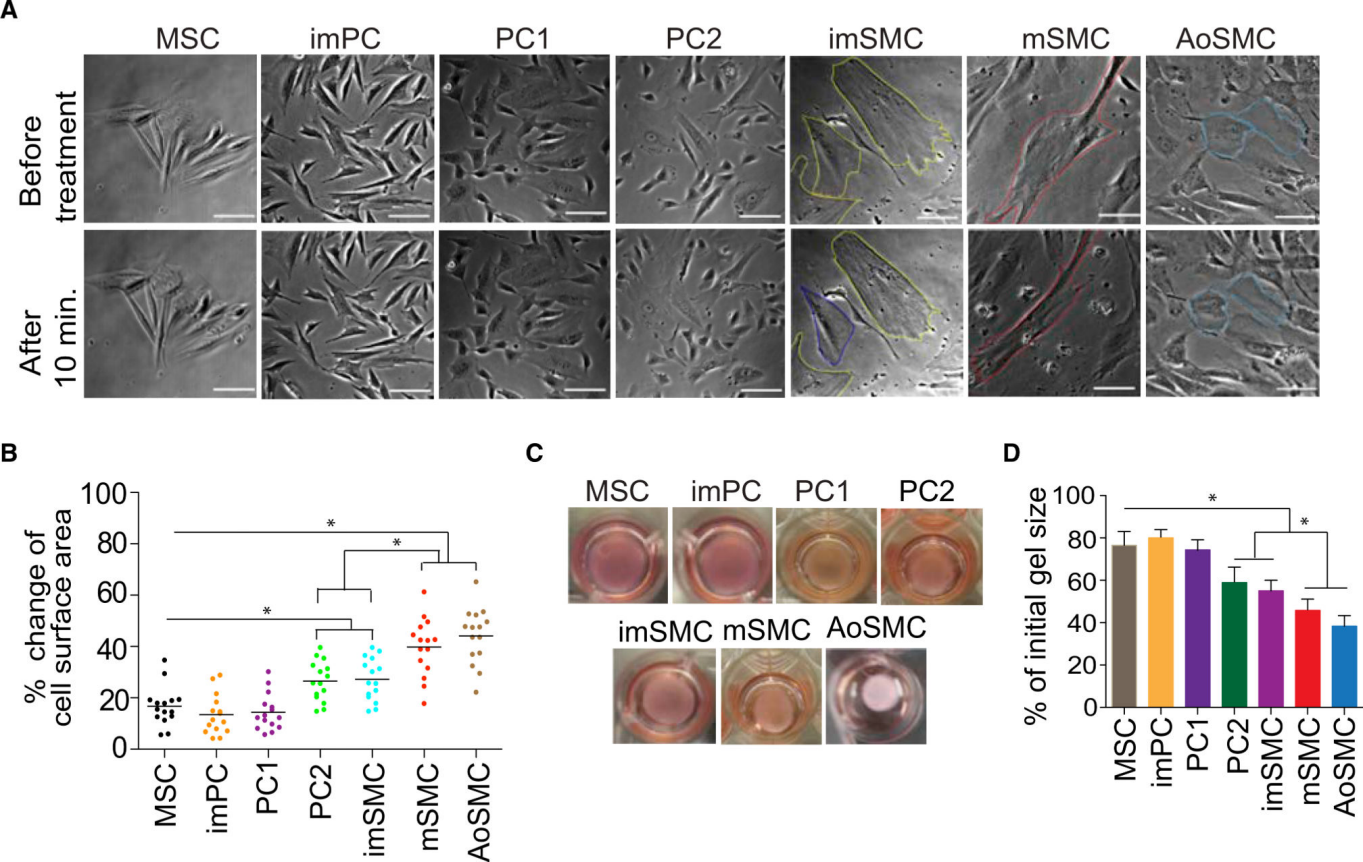
Contractile Properties of MB-Derived Mural Cells and Aortic SMCs (A) Representative images of MB-derived mesenchymal cells and aortic SMCs (aoSMC) (Lonza) before and 15 min after treatment with carbachol (1 μM). Cell cultures were photographed using a Nikon Eclipse Ti-E configured with an A1R confocal system and motorized stage. Scale bar, 50 μm. (B) Changes in individual cell area following treatment with carbachol. Horizontal lines show average changes in the area for all tested 16 cells. (C) Representative images of gel lattices seeded with MSCs, PCs, or SMCs after 48 hr of culture. (D) Assessment of basal contractile tone using collagen gel lattice contraction assay. The changes in lattice area were calculated by dividing the area at 48 hr of culture by the initial area of the lattice. Results are mean ± SE of three independent experiments (*p < 0.01).

To determine the contractile properties of the generated cells, we performed time-lapse studies of individual cells treated with carbachol. Upon this treatment, mSMCs strongly contracted in a tonic fashion and displayed up to a 40% change in surface area (Figures 5A and 5B). The imSMCs and PC2 also contracted following carbachol treatment, but to a lesser degree when compared to mSMCs, whereas MSCs, imPCs, and PC1 presented very limited change in surface area following treatment (Figures 5A and 5B; Movie S4). Similarly, mSMC exhibited the strongest basal contractile tone in the gel lattice assay, with up to a 50% reduction of the initial gel size. The amount of basal tone was minimal in MSCs and imPCs (Figures 5C and 5D). The contractile properties of hPSC-derived mSMCs were comparable to those of early-passage aortic SMCs (Figures 5A–5D).

### In Vivo Vessel-Stabilizing Potential of MB-Derived Mural Cells

**Figure 6.**
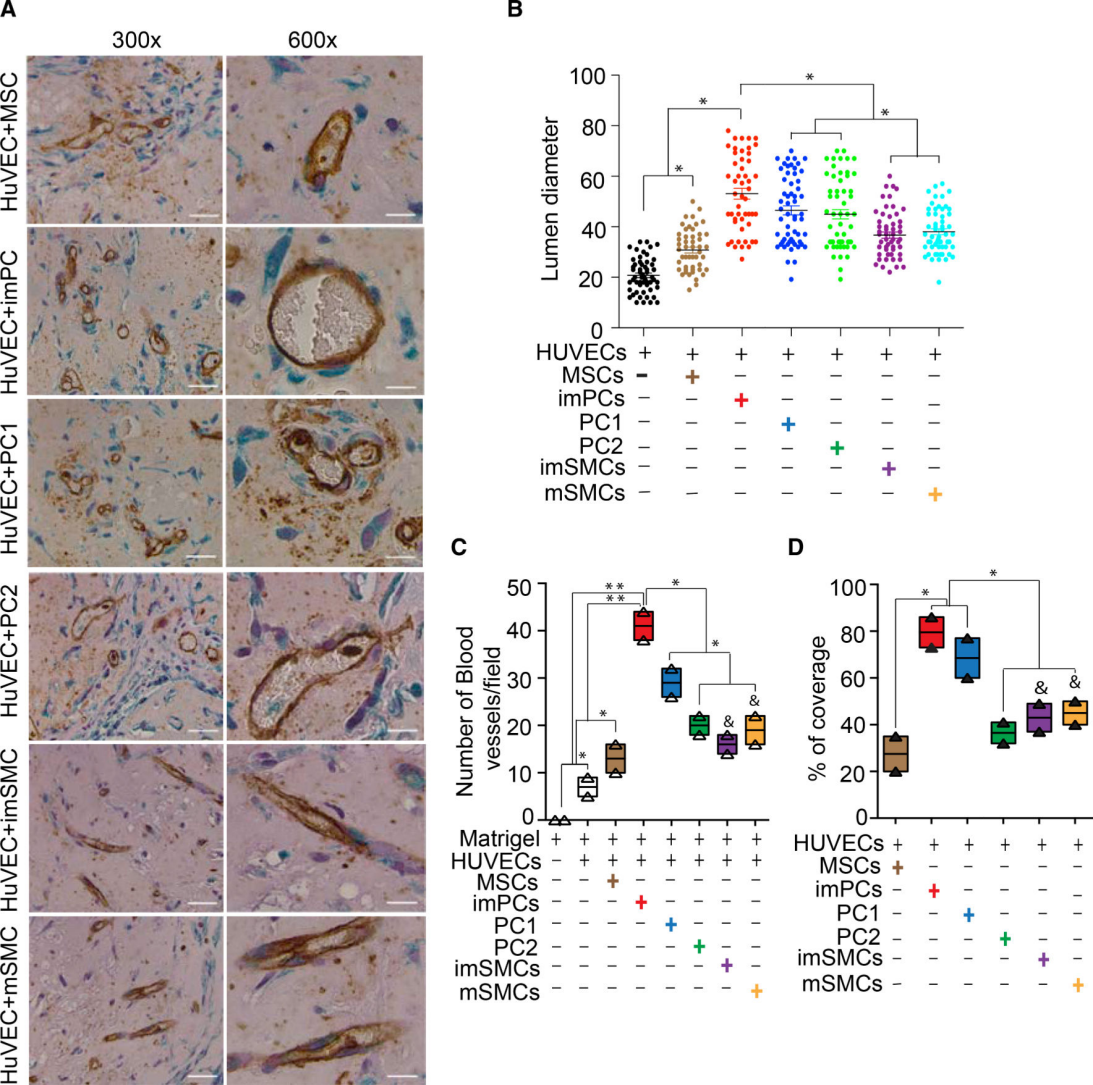
Matrigel Plug Assay Shows the Capacity of MB-Derived Mural Cells to Support the Vasculature Formation In Vivo (A) Representative images of the Matrigel plug stained with anti-human CD31 antibodies (brown) and anti-GFP antibodies (green) following embedding HUVECs with EGFP^+^ mural cells. (B) The scatterplot with mean of lumen diameters. (C) Quantitative analysis of the total number of blood vessels per microscopic field. (D) Quantitative analysis of blood vessel coverage by vasculogenic cells. Results are mean ± SE of three independent experiments (*p < 0.01, **p < 0.001). The ampersand (&) denotes that SMCs support the formation of very small slit-like structures lacking blood cells. In contrast, PCs support the formation of neo-vessels containing blood cells.

To further evaluate the ability of MB-derived vasculogenic cells to support angiogenesis in vivo, we generated GFP-marked mural cells from H9-EGFP hESCs, embedded them in a Matrigel-fibrin matrix with HUVECs, and implanted them into NOD-SCID mice. The formation of human neovasculature was assessed using antibodies to human CD31 to visualize endothelial cells and antibodies to GFP to detect recruited hESC-derived cells. When transplanted alone, HUVECs formed very few small vessels. In contrast, imPCs, PC1, and PC2 strongly supported the formation of neovessels containing circulating blood cells (Figure 6A). Quantification of vessel diameter, density, and percentage of coverage (Figures 6B and 6C) demonstrated that imPCs supported formation of larger vessels and a denser vascular network, whereas fewer and smaller vessels formed in presence of PC1 and especially PC2 (Figures 6A–6C). In contrast, SMCs and MSCs had minimal effects on in vivo vasculature formation from HUVECs. Although we observed an alignment of SMCs and MSCs with CD31^+^ cells, HUVECs implanted with these cells formed very small slit-like spaces that did not contain blood cells (Figures 6A–6D). Thus, these in vivo studies confirmed the superior capacity of in vitro-generated PCs as compared to MSCs and SMCs to support growing vasculature.

## DISCUSSION

During embryonic development, PCs and SMCs originate from the mesenchyme that condenses on the abluminal side of endothelial tube (Hungerford and Little, 1999). The mesenchyme that gives rise to SMCs and PCs originates from different embryonic sources. Lineage-tracing studies have identified at least eight independent sources for vascular SMCs and PCs and highlighted the highly mosaic distribution of SMCs in the vasculature as related to the site of origin (reviewed in Majesky [2007] and Majesky et al. [2011]). The neural crest (Jiang et al., 2000; Le Lièvre and Le Douarin, 1975; Nakamura et al., 2006) and mesoderm (Maeda et al., 2006; Pouget et al., 2008; Waldo et al., 2001) are considered major sources of SMCs and PCs. Within the mesoderm, somitic mesoderm gives rise to vascular SMCs of the trunk, whereas splanchnopleuric mesoderm appears to contribute to mural cells of the viscera (Pouget et al., 2008). Although several studies described generation of PCs from hPSCs (Dar et al., 2012; Orlova et al., 2014), whether these cells originate from mesoderm or neural crest has not been evaluated. It also remains unclear how to distinguish in vitro-generated PCs and SMCs from their progenitors and each other’s because the most reliable criterion for identification of PCs and SMCs in embryonic tissues, the anatomical location (Armulik et al., 2011), cannot be applied to hPSC differentiation studies.

**Figure 7.**
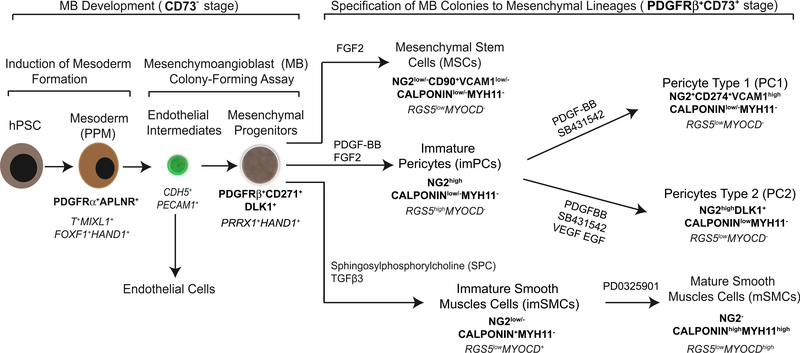
Proposed Model of Mesoderm-Derived Mural Cell Development from hPSCs Distinctive phenotypic and gene (in italics) expression features are shown. Primitive posterior mesoderm (PPM) induced from hPSCs possess a potential to form FGF2-dependent compact spheroid colonies in semisolid medium with mesenchymal and endothelial cell potentials that define MBs. Development of MB colonies proceeds through a core stage at which highly motile PPM cells form clusters of tightly packed endothelial cells (day 3 of clonogenic culture), which subsequently undergo endothelial to mesenchymal transition giving rise to mesenchymal cells. These mesenchymal cells eventually form a shell around the core, resulting in development of spheroid MB colony (day 12 of clonogenic culture) composed of the primitive PDGFRβ^+^CD271^+^CD73^−^ multipotential mesenchymal progenitors. When MB colonies are collected, and cultured in adherent conditions in the presence of the listed factors, they give rise to MSCs, imPCs, and imSMCs. The emerging imPCs could be further specified into CD274^+^ capillary PC1 and DLK1^+^ arteriolar PC2 with pro-inflammatory and contractile phenotype, respectively. Treatment of imSMCs with MEK inhibitor induces their maturation.

To overcome these limitations, we established a PC and SMC differentiation protocol based on our recently identified common mesodermal progenitor for endothelial cells and MSCs, MB (Vodyanik et al., 2010). MB arises at primitive streak stage of development from APLNR^+^PDGFRα^+^CD31^−^VE-cadherin^−^ primitive posterior mesoderm and distinct from the vascular progenitor with primary endothelial characteristics mesoangioblast, which expresses Flk1, CD31, CD34, and VE-cadherin endothelial markers, and was found in mouse aorta at embryonic day 9.5 (E9.5) (Minasi et al., 2002). Using functional and phenotypic analysis in conjunction with gene expression profiling of clonally derived MB and its progeny, we were able to identify the lineage tree and specific markers of progenitors leading to the development of PCs and SMCs from mesoderm. As shown in Figure 7, hPSC-derived APLNR^+^PDGFRα^+^ mesodermal cells placed in serum-free semisolid medium with FGF2 upregulate *CDH5* and *PECAM1* expression and form clusters of tightly packed cells (cores) with angiogenic potential on day 3 of clonogenic culture. Subsequently, core-forming endothelial intermediates undergo endothelial-mesenchymal transition giving rise to mesenchymal cells, which form a shell around the core, resulting in formation a fully developed MB colony on day 12 of clonogenic culture (Slukvin and Vodyanik, 2011; Vodyanik et al., 2010). The sequence of events leading to the formation of MB colonies closely recapitulates the events leading to angioblast formation in vivo. In chicken embryo, FGF produced by endodermal cells induces the aggregation of migrating posterior cells adjacent to the endoderm, upregulation of KDR, and the formation of angioblasts (Flamme et al., 1995; Risau and Flamme, 1995). The development of mesenchyme from primitive posterior mesoderm through endothelium (specialized squamous epithelium) also follows the common principle of mural cell development in the embryo from epithelium. The origin of the majority of mural cells in heart, lung, gut, and liver has been mapped to mesothelium, a specialized squamous epithelium that lines coelomic cavities and organs (Asahina et al., 2011; Cai et al., 2008; Que et al., 2008; Wilm et al., 2005). Several lines of evidence also indicate that the certain mural cells originate from endothelium (Armulik et al., 2005; de Lange et al., 2004; Moonen et al., 2010).

The MB colony formed in clonogenic medium is composed of immature PDGFRβ^+^ CD271^+^CD73^−^ embryonic mesenchyme with the potential to generate MSCs, PCs, and SMCs following culture with FGF2, PDGF-BB plus FGF2, or SPC plus TGFβ3, respectively. PCs induced with PDGF-BB can be further specified into capillary CD274^+^ PC1 or arteriolar DLK1^+^ PC2 exhibiting a proinflammatory or contractile phenotype, respectively. Induction of SMC maturation was achieved with MEK inhibitor.

Interestingly, we found that CD146 commonly used to identify somatic PCs is expressed at all stages of mesenchymal cell development, including MB colonies, MSCs, PCs, and SMCs, and therefore has little value to discriminate the progenitor stage and different types of mural cells generated from hPSCs. We also noticed that SMA (ACTA2) mRNA was expressed at early mesodermal stages of development, endothelial progenitors and early blood cells (Vodyanik et al., 2010), which is consistent with observation of SMA expression in cultured endothelial cells from mouse embryo (Pouget et al., 2008). Therefore, we used molecular profiling of the hPSC-derived cells to select a set of markers enabling the reliable separation of the different stages of mural cell development. As summarized in Figure 7 and Table S1, we revealed that a PDGFRβ^+^CD271^+^CD73^−^ phenotype along with unique expression of *PRRX1* and *HAND1* genes could distinguish primitive mesenchymal progenitors within MB colony from downstream progeny. The MSCs can be discriminated from mesenchymal progenitors, PCs and SMCs, based on the expression of CD90, CD73, and CD105 and low/lack expression of VCAM, and typical SMC and PC markers. High expression of calponin and SMA, along with the unique expression of *MYOCD*, discriminates SMCs from mesenchymal precursors and PCs. The detection of MYH11 is a strong indicator of SMC maturation. The NG2^+^Calponin^low/−^ phenotype, coupled with a lack of *MYOCD* expression, separates PCs from other types of cells. Capillary proinflammatory PC1 was uniquely identified by the expression of CD274 (PDL1) and high expression of VCAM1, whereas arteriolar contractile PC2 uniquely expresses DLK1 along with CD73. The set of additional subset-specific genes is listed in Table S1.

Overall, the lineage-tree and lineage-specific markers of mesenchymal and vasculogenic cells identified in the current study provide a platform for the interrogation of the molecular mechanisms leading to mural cell development from hPSCs and modeling genetic diseases associated with vascular and skeletal abnormalities by employing patient-specific iPSCs. In addition, these studies provide a reproducible method for the scalable generation of distinct populations of PCs and SMCs of mesodermal origin from hPSCs for potential applications in regenerative medicine.

## EXPERIMENTAL PROCEDURES

### Maintenance and Differentiation of hPSCs

The hESC H1 and H9-EGFP lines, fibroblast-derived hPSC line DF-19–9-7T, and blood-derived hPSC line IISH2i-BM9 were obtained from WiCell Research Institute. Mouse OP9 bone marrow stromal cell line was provided by Toru Nakano (Osaka University, Osaka, Japan). hPSCs were maintained on irradiated mouse embryonic fibroblasts as described previously (Yu et al., 2007) and induced to differentiate in coculture with OP9 stromal cells (Vodyanik and Slukvin, 2007). In some experiments, hPSCs were maintained in chemically defined conditions (Chen et al., 2011) and induced to differentiate into mesoderm with BMP4, Activin A, and FGF2 (all from Peprotech) in chemically defined conditions (Uenishi et al., 2014). All cells were cultured in humidified incubators, with atmospheres at 37°C and 5% CO_2_.

### Colony-Forming Culture for MBs

A single-cell suspension of day 2 differentiated cells was prepared at 0.5–2 × 10^4^ cells/mL in a semisolid colony-forming serum-free medium (CF-SFM) with 20 ng/mL FGF2 (Vodyanik et al., 2010). Individual MB colonies were picked from culture on day 12 under the inverted microscope. For bulk collection of MB colonies (>100 μm in diameter), day 12 colony-forming cultures were diluted 1/5 in DMEM/F12 medium and filtered through 100-μm cell strainers (BD Biosciences).

### MB Colony-Derived PCs

Individual or multiple MB colonies collected by filtration (>100 colonies per culture) were plated onto culture dishes pre-coated with human fibronectin (3 μg/mL; BD Biosciences) and human collagen I (10 μg/mL; BD Biosciences) in mesenchymal serum-free expansion medium (M-SFEM) containing 10 ng/mL FGF2 (Vodyanik et al., 2010) with added 50 ng/mL PDGF-BB. After 3 days, the attached colonies were dissociated by StemPro Accutase solution (Invitrogen) and plated on the fibronectin/collagen-coated plates in commercial Pericyte Medium (ScienCell Research Laboratories) for 14 days until the cells formed a confluent monolayer. The first confluent culture at this time point was labeled as immature PCs (imPCs) and denoted as passage 1. Colonyderived PC lines were routinely maintained by 3-day subculture in commercial Pericyte Medium on fibronectin/collagen-coated plates using StemPro Accutase detachment solution. For maturation, imPCs were cultured on fibronectin/ collagen-coated plates in Pericyte Medium with added SB431542 (10 μM) and PDGF-BB (50 ng/mL) or SB431542 (10 μM), PDGF-BB (10 ng/mL), VEGF (10 ng/mL), and EGF2 (2 ng/mL) for 6 days to generate capillary PCs (PC1) and arteriolar PCs (PC2), respectively. PC1 and PC2 cultures were dissociated by StemPro Accutase solution, split at 1:5 in Pericyte Medium, and cultured on the fibronectin/collagen-coated dishes for an additional 4 days.

### MB Colony-Derived SMCs Lines

Individual or multiple MB colonies collected by filtration (>100 colonies per culture) were plated on human fibronectin (3 μg/mL; BD Biosciences) and human collagen (10 μg/mL; BD Biosciences)-coated plates in complete EGM-2 medium (Lonza) with added SPC (2 μM) and TGFβ3 (2 ng/mL). After 6 days, the attached colonies were dissociated by StemPro Accutase solution (Invitrogen) and cultured on fibronectin/collagen-coated plates in complete EGM-2 medium (Lonza) without SPC and TGFβ3 for 14 days. The first confluent culture at this time point was labeled as immature or proliferative SMCs (imSMCs) and denoted as passage 1. imSMCs were maintained by 5-day subculture in EGM-2 medium on fibronectin/collagen-coated plates. The SMCs were matured by culturing in complete EGM-2 medium (Lonza) with added PD0325901 MEK inhibitor (1 μM) for an additional 6 days.

### MB Colony-Derived MSC Lines

MSC lines were established from MB colonies by culture of colonies on collagen/fibronectin-coated plates in M-SFEM medium with 10 ng/mL of FGF2 (Vodyanik et al., 2010).

### Mice

All animal procedures were performed under protocols approved by University of Wisconsin Institutional Animal Care and Use Committee.

## Supplementary Material

Data S1

Data S2

Data S3

Movie S1

Movie S2

Movie S3

Movie S4

Supplemental

## Highlights

- Mesodermal mural cells originate from a clonal precursor mesenchymoangioblast (MB)
- PDGFRβ^+^CD271^+^DLK1^+^CD73^−^ phenotype identifies intermediate mural cell precursors
- MB-derived pericytes can be specified to capillary and arteriolar types
- Lineage tree of vasculogenic progenitors in hPSC cultures is proposed
