# Supplementary material for: Specification and Diversification of Pericytes and Smooth Muscle Cells from Mesenchymoangioblasts
Source: Cell Rep. Author manuscript; Available in PMC 2019 Mar 21. (PMC6428685; doi:10.1016/j.celrep.2017.05.019)
Supplement: Supplemental [file NIHMS1003476-supplement-Supplemental.pdf]

Cell Reports, Volume *19*

Supplemental Information

Specification and Diversification of Pericytes and Smooth Muscle Cells from Mesenchymoangioblasts

Akhilesh Kumar, Saritha Sandra D'Souza, Oleg V. Moskvin, Huishi Toh, Bowen Wang, Jue Zhang, Scott Swanson, Lian-Wang Guo, James A. Thomson, and Igor I. Slukvin

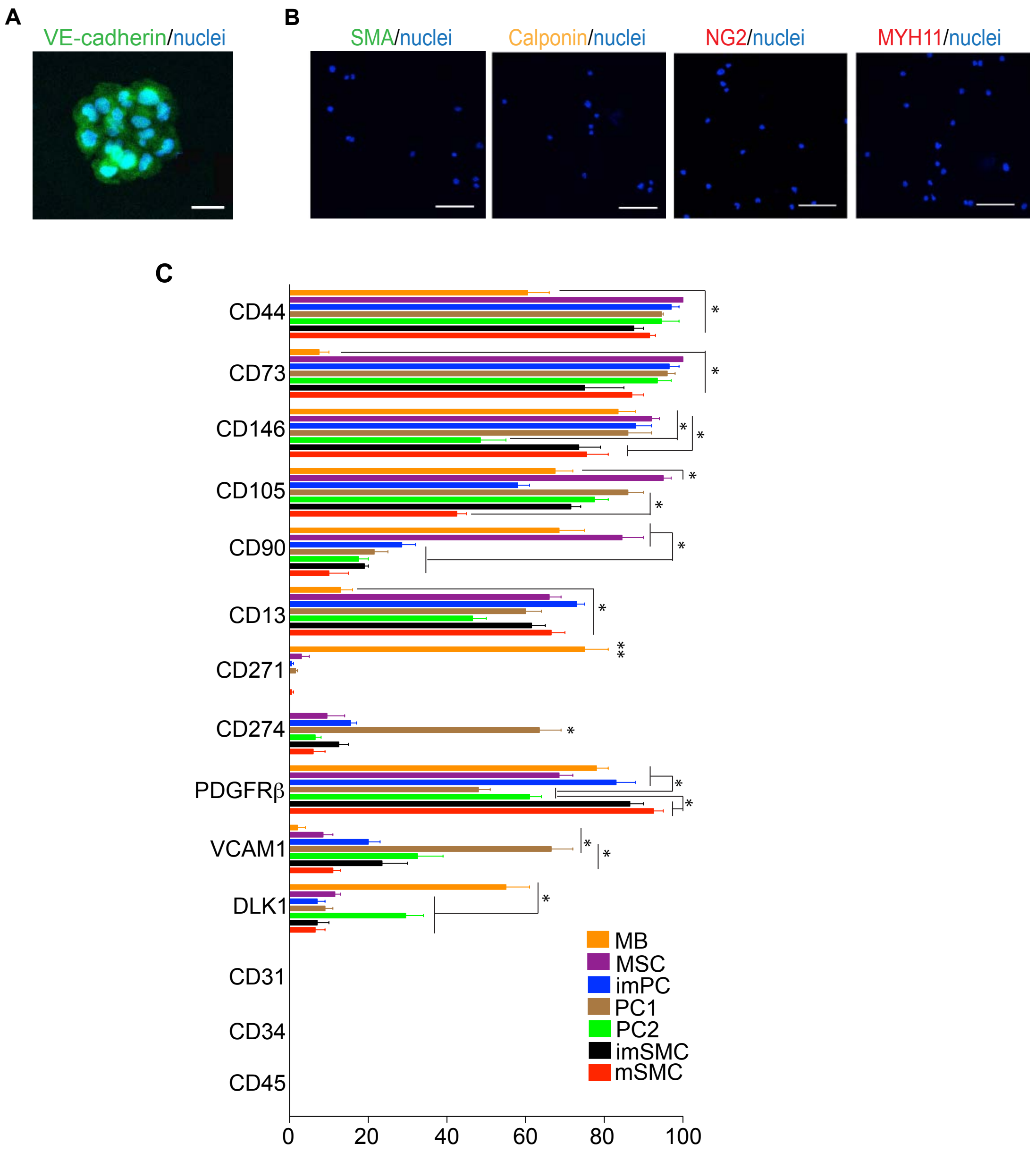

Figure S1. Immunofluorescent and flow cytometry analysis of MB colonies, Related to Figure 1. (A) Cytospins from cores formed on day 4 of clonogenic culture were prepared and stained with VE-cadherin antibodies and then photographed. Scale bar is 10 μm. (B) Single cell suspension from MB colonies was generated and used to prepare cytospins. Cytospins were stained with SMA, Calponin, MYH11 and NG2 antibodies and then photographed. Mesenchymal cells composing MB colonies (MB) are negative for PC and SMC markers. Scale bar is 50 μm. (C) Summary of phenotype of MB-derived mural cells, as detected by flow cytometry. Results are displayed as mean ± SE of three independent experiments (*$p<0.01$, **$p<0.001$).

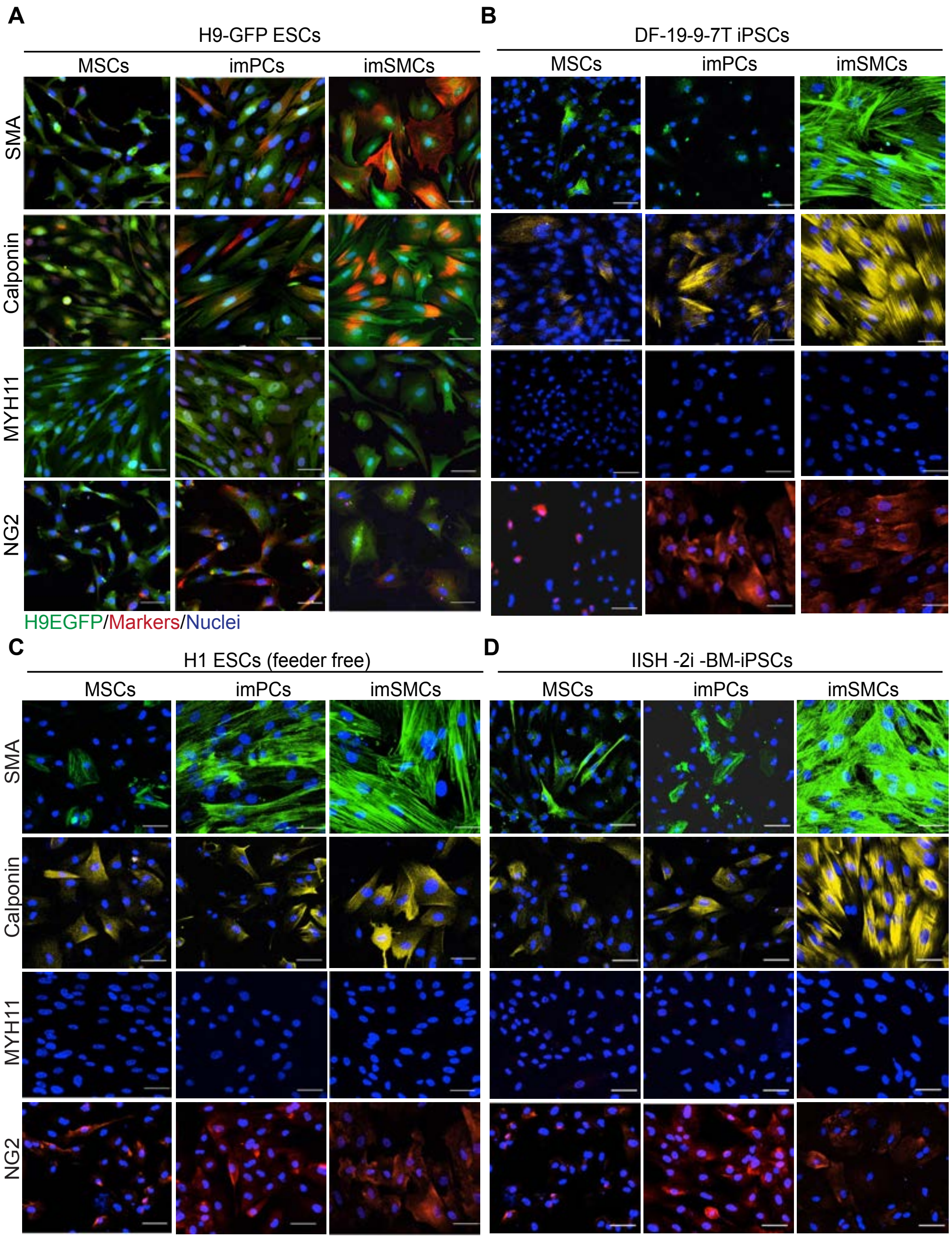

Figure S2. Immunohistochemistry analysis of SMC and PC markers in mural cells generated from different hPSC lines through the MB pathway, Related to Figure 1. (A) Mural cells were generated from H9-EGFP hESCs differentiated in coculture with OP9. (B) Mural cells were generated from transgene-free DF-19-9-7T iPSCs differentiated in coculture with OP9. (C) Mural cells were generated from H1 hESCs differentiated in chemically defined conditions. (D) Mural cells were generated IISH2i-BM9 blood-derived iPSCs differentiated in defined conditions. The hPSC lines have the capacity to generate NG2^{high}Calponin$^{low/-}$ imPCs and NG2$^{low/-}$Calponin^{high} imSMCs from MBs induced in OP9 coculture or in chemically defined conditions.

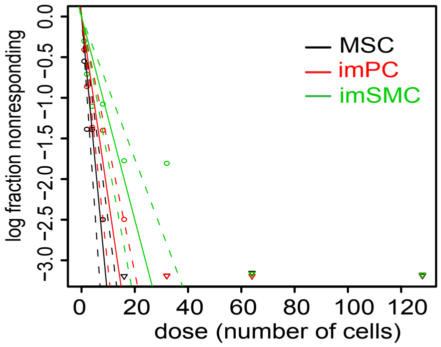

Frequency (95% confidence interval)

Group	Lower	Estimate	Upper
MSC	3.98	2.84	2.1
imPC	6.34	4.48	3.22
imSMC	11.43	8	5.65

Figure S3. Limiting dilution assay to determine a frequency of PC, SMC and MSC progenitors within MB colonies, Related to Figure 1.

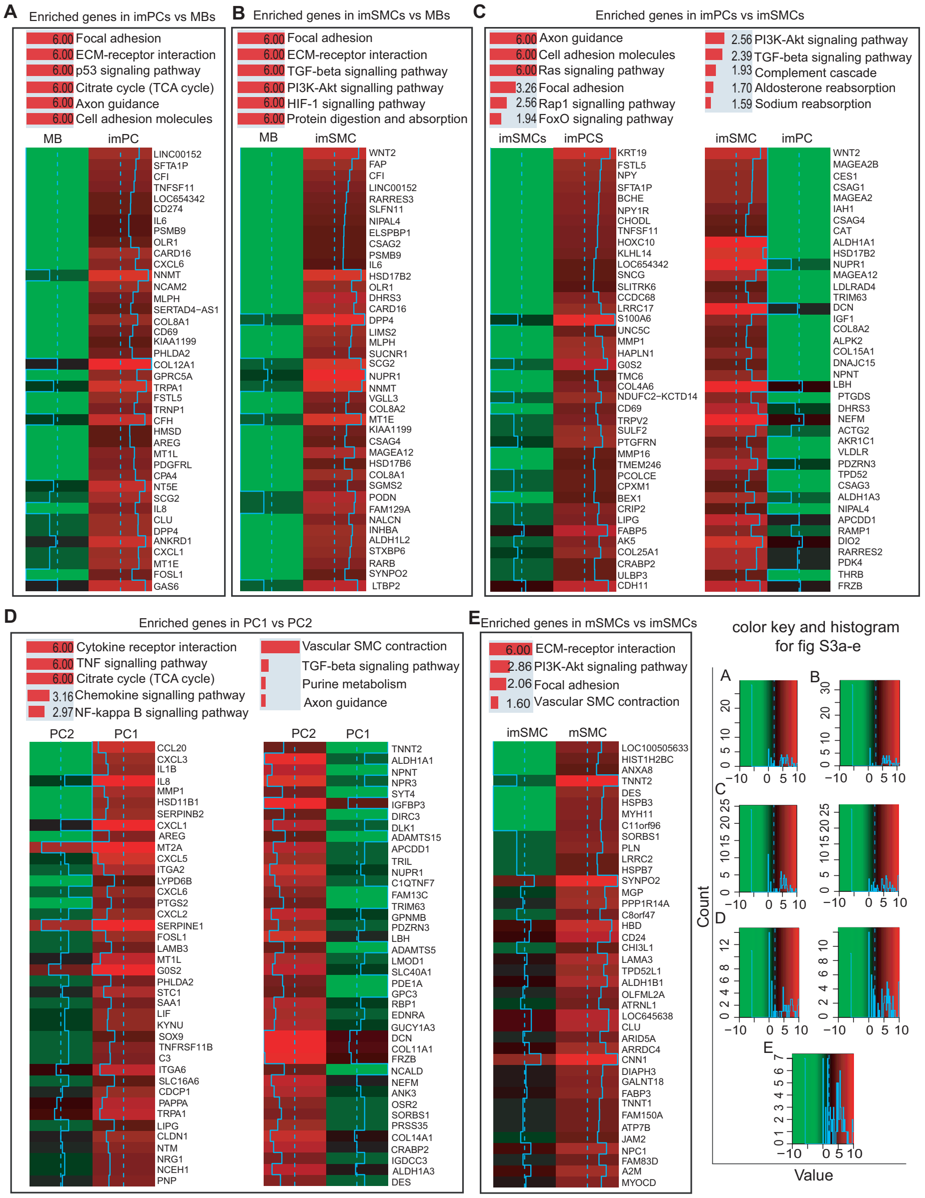

Figure S4. Comparative analysis of differentially expressed genes in *in vitro* generated mural cells, Related to Figure 3. (A) Genes upregulated in imPCs versus MB colonies. (B) Genes upregulated in imSMC versus MB colonies. (C) Genes differentially expressed imPCs versus imSMCs. (D) Genes differentially expressed in PC2 versus PC1. (E) Genes differentially expressed in mSMCs versus imSMCs. The classification of differentially expressed into functional categories defined by KEGG pathways. Enrichment scores are expressed as $-\log_{10}$(FDR). Red indicates terms associated with upregulated genes; green indicated terms associated with downregulated genes. All shown terms have a probability value <0.05. Heat maps display the top 40 overexpressed genes ranked by the fold change in their expression in the target cell type. Heatmap coloring is done by expression value. At least tenfold difference in expression was estimated in the tpm values for the shown genes. Heat maps shows mean of three independent experiments with hPSC_derived mural cells, two independent experiments with aortic SMCs, and RNAseq data of somatic PCs from single experiment.

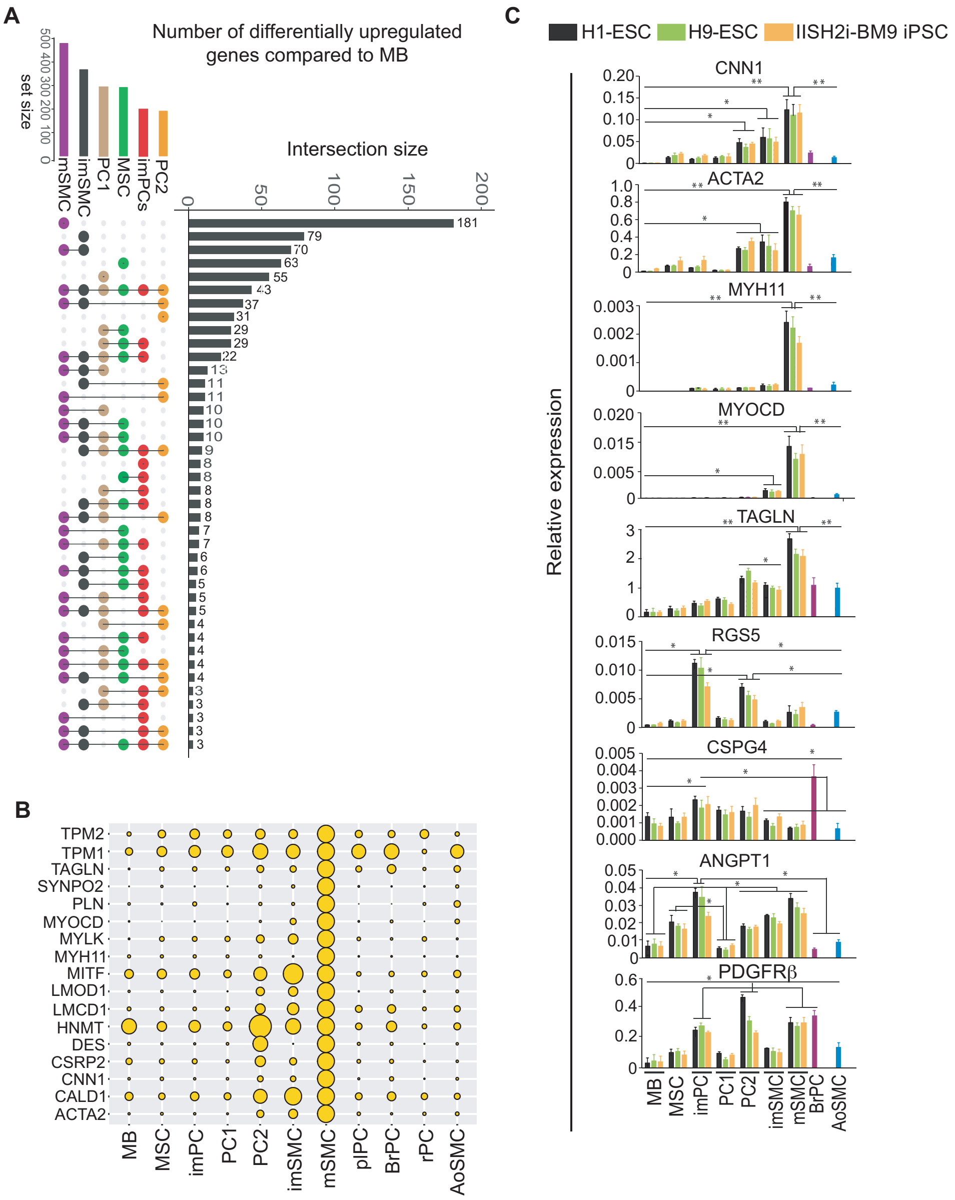

Figure S5. Analysis of gene expression in MB-derived mural cells, Related to Figure 3. (A) Bar chart illustrating multi-set intersections of genes selected as upregulated in the target cell type (relative to MB colonies) according to the rules for marker gene selection described in the Methods, visualized via UpSet algorithm(Lex et al., 2014). The combination matrix marked with colored circles identifies the genes uniquely upregulated by cell subset/subsets (intersections), while the overlying bars encode the set size and bars on the right show total number of genes uniquely upregulated. (B) Balloon graph shows the relative expression of typical SMC genes as compared to mSMCs (indicated population/mSMC ratio). The balloon area in mSMCs corresponds to mSMC/mSMC ratio of 1. Results are mean of three independent experiments with hPSC_derived mural cells, two independent experiments with aortic SMCs, and from single RNAseq somatic PCs experiment. (C) Quantitative qPCR analysis of representative transcripts in the indicated mesenchymal cell populations generated from H1 and H9-GFP hESCs, and blood-derived IISH2i-BM9 iPSCs. Scale bars represent a mean ± SE of three experiments normalized to *GAPDH* levels (**p<0.001, *p<0.01).

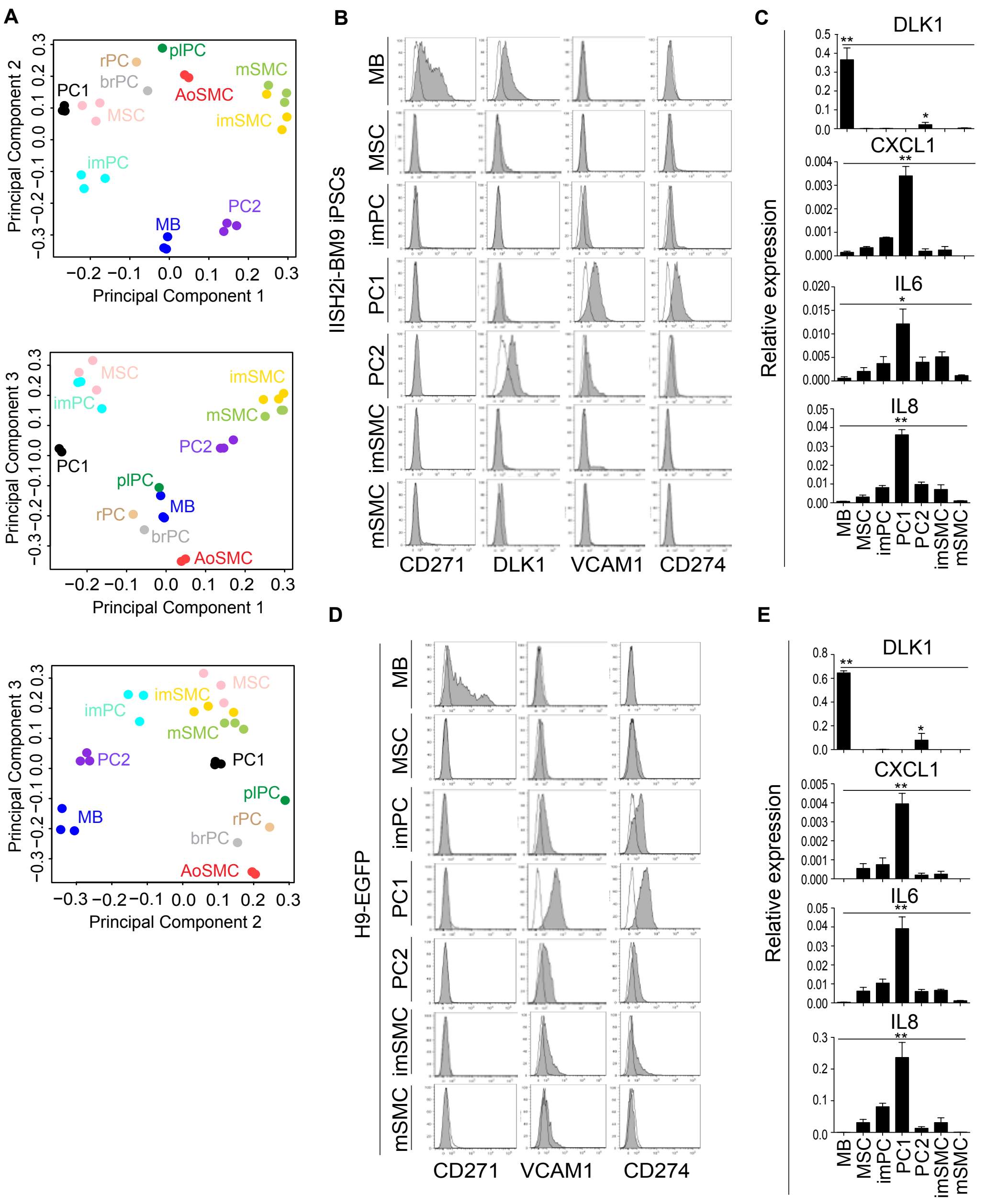

Figure S6. PCA, flow cytometric and qPCR analysis of MB-derived mesenchymal cell populations, Related to Figure 3. (A) PCA of transcriptome data for the 11 cell types (points are colored according to individual sample label). 2D PCA plots are shown. plPC placental, brPC brain, rPC retinal PCs, aoSMC aortic SMCs. (B) Flow cytometric and (C) qPCR analysis of subset-specific markers in mesenchymal cell subsets generated from blood-derived IISH2i-BM9 iPSCs. (D) Flow cytometric and (E) qPCR analysis of subset-specific markers in mesenchymal cell subsets generated from blood-derived H9-EGFP hESCs. Flow cytometric and qPCR analysis confirms expression of subset-specific markers in MB-derived mesenchymal cell populations from H9 hESCs and blood derived iPSCs. Results shown are mean ± SE of three independent (**p<0.001, *p<0.01).

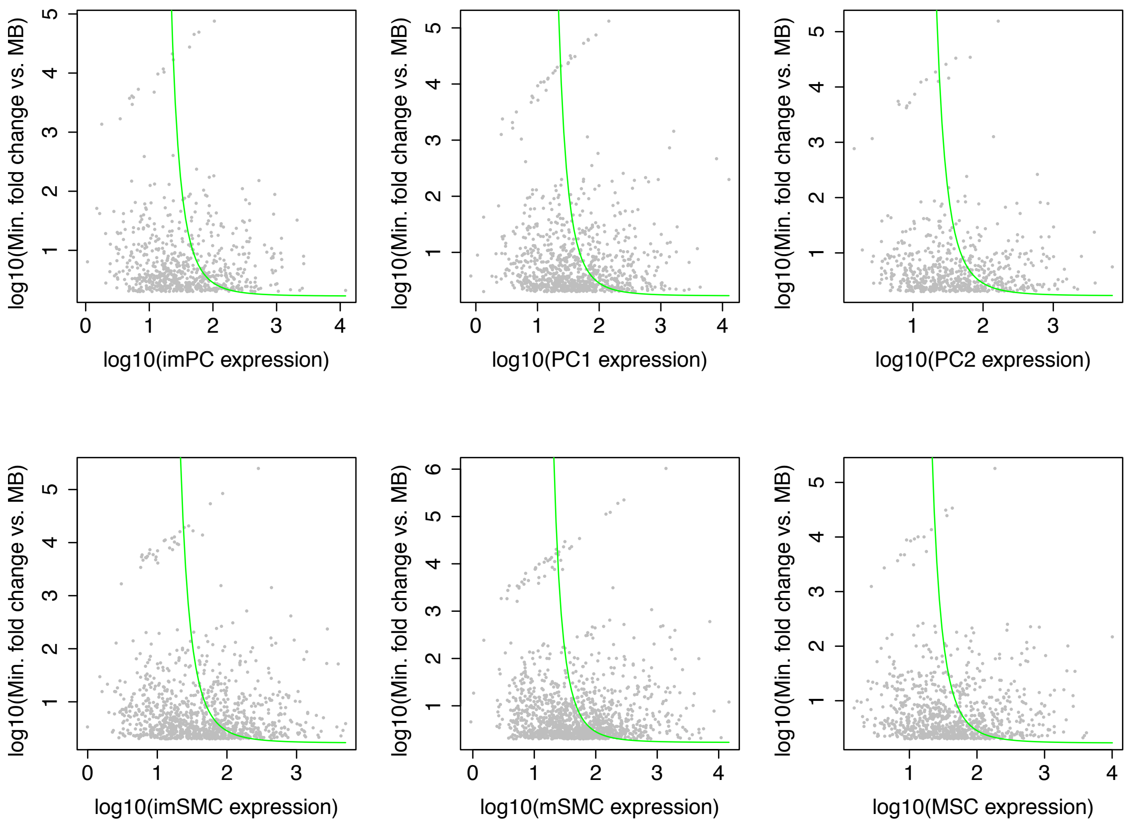

Figure S7. Application of the hyperbolic-exponential filter (see the "marker selection" section in the Experimental Procedures) to apply adaptive fold change cutoff that depends on the gene expression value in the target cell type (MB case is shown), Related to Experimental Procedure. The same filter was consistently applied to select set of markers for all the 6 cell types indicated along the X axes.

Table S1. Phenotypic and functional properties of hPSC-derived mesenchymal progenitors and mural cells, Related to Figure 7.

Cell Type	Distinct phenotype	Mesenchymal/ Endothelial	Other PC and SMC markers	Uniquely Expressed Genes	Support of Vessel Formation		Contractile Properties
					In vitro	*In vivo*	
Primitive Posterior Mesoderm	APLNR^{+}PDGFRα^{+}	PDGFRβ^{-} CD73^{-}CD105^{-} CD31^{-}	N/A	*T, MIXL1, EOMES, HAND1*	N/A	N/A	N/A
MB Colonies	PDGFRβ^{+}CD271^{+} CD73^{-}	PDGFRβ^{+} CD146^{+}CD73^{-} CD105^{low}CD31^{-}	*RGS5^{low} ACTA2^{low} MYOCD^{-}*	*PRRX1, HAND1,TBR1, LHX1, EMCN*	N/A	N/A	N/A
MSCs	NG2$^{low/}$CD90^{+} VCAM1$^{low/-}$Calponin$^{low/-}$ Desmin^{-}MYH11^{-}	PDGFRβ^{+} CD146^{+}CD73^{+} CD105^{+}CD31^{-}	*RGS5^{low}, ACTA2^{low} MYOCD^{-}*	*MAMDC2, GRIA1*	Minimal	Low density, small vessels	Minimal
imPCs	NG2^{high}Calponin$^{low/-}$ Desmin$^{low/-}$MYH11^{-}	PDGFRβ^{+} CD146^{+}CD73^{+} CD105^{low}CD31^{-}	*RGS5^{high} ACTA^{low} MYOCD^{-}*	*FSTL5, PDGFRL*	Strong up to 72 hours	High density, large diameter vessels	Minimal
PC1 capillary/ pro-inflammatory	NG2^{+}CD274^{+} VCAM1^{high}Calponin$^{low/-}$ Desmin$^{low/-}$MYH11^{-}	PDGFRβ^{+} CD146^{+}CD73^{+} CD105^{+}CD31^{-}	*RGS5^{low} ACTA2^{low} MYOCD^{-}*	*IL8, CXCL1, CXCL2,CXCL3, CXCL5,CCL20, IL6, IL1B*	Strong up to 7 days	High density, intermediate diameter vessels	Minimal
PC2 arteriolar/ contractile	NG2^{high}DLK1^{+} Calponin^{low}Desmin^{+} MYH11^{-}	PDGFRβ^{+} CD146^{+}CD73^{+} CD105^{+}CD31^{-}	*RGS5^{+} ACTA2^{intrmd} MYOCD^{-}*	*FRZB, NPR3, EDNRA*	Strong up to 7 days	Low density, intermediate diameter vessels	++
imSMCs proliferative/ synthetic	NG2^{low}Calponin^{+} Desmin^{low}MYH11^{-}	PDGFRβ^{+} CD146^{+}CD73^{+} CD105^{+}CD31^{-}	*RGS5$^{low/-}$ ACTA2^{high} MYOCD^{+}*	*DIO2, IGF1, HSD17B2*	Align to tubes and pull tubes apart	Low density, slit-like spaces	++
mSMC contractile	NG2^{low}Calponin^{high} Desmin^{high}MYH11^{+}	PDGFRβ^{+} CD146^{+}CD73^{+} CD105^{+}CD31^{-}	*RGS5^{low}, ACTA2^{high} MYOCD^{high}*	*TNNT2^{high}, SYNPO2^{high}*	Align to tubes and pull tubes apart	Low density, slit-like spaces	+++

Table S2. List of antibodies used in this study, Related to Experimental Procedure.

Antigen	Label	Clone	Dilution	Application	Company	Cat. No.
Mouse IgG k Isotype control	FITC	MOPC-21	1/30	FACS	BD Bioscience	554679
Mouse IgG k Isotype control	PE	MOPC-21	1/30	FACS	BD Bioscience	554680
Mouse IgG k Isotype control	APC	MOPC-21	1/30	FACS	BD Bioscience	554681
Anti-human CD13	FITC	123H1	1/30	FACS	BD Bioscience	M101-4
Anti-human CD31	FITC	WM59	1/20	FACS	BD Bioscience	555445
Anti-human CD34	PE	8G12	1/20	FACS	BD Bioscience	348057
Anti-human CD45	APC	HI30	1/20	FACS	BD Bioscience	555485
Anti-human CD73	PE	AD2	1/20	FACS	BD Bioscience	550257
Anti-human CD90	APC	5E10	1/200	FACS	BD Bioscience	559869
Anti-human CD105	PE	SN6	1/50	FACS	Caltag-invitrogen	MHCD10504
Anti-human CD146	PE	P1H12	1/20	FACS	BD Bioscience	550315
Anti-mouse CD29	PE	HM beta 1-1	1/50	FACS	AbD Serotec	MCA2298
Anti-human CD29	PE	MEM-101A	1/50	FACS	Caltag-Invitrogen	CD2904
Anti-human CD271	APC	ME20.4-1.H4	1/50	FACS	Miltenyi Biotech	120-002-230
Anti-human CD44	APC	G44-26	1/50	FACS	BD Bioscience	559942
Anti-human CD274	PE	29E.2A3	1/50	FACS	BioLegend	329705
Anti-human APLNR	APC	72133	1/50	FACS	R&D Systems	FAB856A
Anti-human DLK1	Alexa Fluor®488	211309	1/20	FACS	R and D system	FAB1144G-025
Anti-human VCAM1	APC	51-10C9	1/50	FACS	BD Bioscience	551147
Anti-human ICAM1	PE	HCD54	1/50	FACS	BioLegend	322702
Anti-human PDGFRα	PE	αR1	1/30	FACS	BD Bioscience	556002

Anti-human PDGFRβ	PE	28D4	1/30	FACS	BD Bioscience	558821
Anti-human NG2	None	Polyclonal	1/20	IF	eBioscience	14-6504
Anti-human NG2	None	Polyclonal	1/200	IF	EMD Millipore	MAB5384
Anti-human ASMA	None	1A4	1/100	IF	Abcam	Ab7817
Anti-human MYH11	None	SMMS-1	1/100	IF	Abcam	ab106919
Anti-human Calponin	None	CALP	1/1000	IF	Thermo Scientific™	MS-1168-PO
Anti-human Desmin	None	Polyclonal	1/1000	IF	Thermo Scientific™	Rb-9014-PO
Anti-GFP	None	Polyclonal	1/100	IF	Novus Biologicals	NB600-303
Anti-human CD31	None	JC/70A	1/100	IF	Thermo Scientific™	MS-353-S0
IgG rabbit	None	Polyclonal	1/100	IF	EMD Millipore	NI01-100UG
IgG mouse	None	Polyclonal	1/50	IF	EMD Millipore	NI03-100UG
Donkey Anti –mouse IgG (H+L)	Alexa Fluor®488	Polyclonal	1000	IF	Invitrogen	A-21206
Donkey Anti –Rabbit IgG (H+L)	Alexa Fluor®555	Polyclonal	1/1000	IF	Invitrogen	A-21422

Table S3. List of primers used for qRT-PCR, Related to Experimental Procedure.

Gene	Direction	Sequences
ACTA2	Forward	5’ GTG TGC CCC TGA AGA GCA T 3’
	Reverse	5’ GCT GGG ACA TTG AAA GTC TCA 3’
CSPG4	Forward	5’ GTC TTT TGA GGC TGC CTG TC 3’
	Reverse	5’ CTG TGT GAC CTG GAA GAG CA 3’
PDGFRB	Forward	5’ TGC AGC ACC ACT CCG ACA AGC 3’
	Reverse	5’ TCG CTC TCC CCG GTC AAG GAC 3’
CALD1	Forward	5’ CTG GCT TGA AGG TAG GGG TTT 3’
	Reverse	5’ TTG GGA GCA GGT GAC TTG TTT 3’
RGS5	Forward	5’ TCC AGG GAA TCA CGC CAC TGC 3’
	Reverse	5’ AGC CAG ACT CAG TTG GTG ACC T 3’
MYCOD	Forward	5’ CTC GGC TTC CTT TGA ACA AG 3’
	Reverse	5’ CTT CCC AGA GAA TCC ATC CA 3’
MYH11	Forward	5’ GGA GGA TGA GAT CCT GGT CA 3’
	Reverse	5’ TTA GCC GCA CTT CCA GTT CT 3’
CNN1	Forward	5’ CAA CCA CCA CGC ACA CAA CTA C 3’
	Reverse	5’ GGT CCA GCC AAG AGC AGC AG 3’
IL6	Forward	5′-GGTACATCCTCGACGGCATCT-3′
	Reverse	5′-GT GCCTCTTTGCTGCTTTCAC-3′
IL8	Forward	5'-ACTGAGAGTGATTGAGAGTGGAC-3'
	Reverse	5'-AAC CCT CTG CAC CCA GTT TTC-3
DLK1	Forward	5’-AAG GAC TGC CAG AAA AAG GAC-3’
	Reverse	5’-GCA GAA ATT GCC TGA GAA GC-3’.
CXCL1	Forward	5'-GCCCAAACCGAAGTCATAGCC-3'
	Reverse	5'-ATCCGCCAGCCTCTATCACA-3'

Supplementary Experimental Procedures

Evaluation of frequency of MB-derived mesenchymal cells

To evaluate the frequency of MB-derived cells extreme limiting dilution assay (ELDA) was performed as described previously (Hu and Smyth, 2009). Briefly, individual MB colonies were collected and induced in PC, SMC, and MSC conditions and after three days cells were harvested and plated in decreasing numbers from 128 cells/well to 1cell/well in 100 µl of media. Cultures were maintained until day 14, when the number of wells containing vasculogenic cells for each cell plating density were recorded, calculated and plotted using an ELDA analysis program available on line (http://bioinf.wehi.edu.au/software/elda).

Evaluation of the proliferative potential of MB-derived mesenchymal cells

Cell cultures were maintained at a plating density of 10^4 cells/cm^2 and harvested at near total confluence. Doubling time at each passage was calculated by the following: dT (hours) = ln (2) x duration of culture (hours) / ln (output cell number / input cell number). The cumulative number of cells at each passage was calculated using: N p(n) = N p(n-1). (output N p(n) / input N p(n)), with N representing cell number, p(n-1) – previous passage, p(n) – present passage.

Fluorescence Activated Cell Sorting (FACS) analysis

For flow cytometary analysis, cells were disscociated with accutase and single- cell suspensions were prepared in FACS buffer. Cell-surface staining was completed using the antibodies outlined in Table S2. 7-aminoactinomycin D (7AAD) was used for dead cell exclusion, as described (Vodyanik and Slukvin, 2007). Cells were analyzed with a FACSCalibur flow cytometer (BD Biosciences). Control staining with the appropriate isotype matched mouse monoclonal antibody controls was included to establish a threshold for positive staining and subset gating.

Immunofluorescence

Cells were prepared for immunofluorescence as previously described (Vodyanik et al., 2010). Briefly, cells were fixed for 15 minutes, washed with phosphate buffered saline (PBS), permeabilized with a solution of 0.1% Triton-X (Sigma) for ten minutes, washed with PBS, and incubated overnight with anti-human SMA (1:100; Abcam), anti-human NG2 (1:100; Millipore), anti-human MYH11 (1:300; Abcam), anti-human Calponin (1:1000; Thermo Fisher) and anti-human Desmin (1:1000; Thermo Fisher). Cells were fixed with 4% paraformaldehyde for all antibodies except MYH11, wherein cells were fixed with methanol. Cells were washed five times with PBS-Tween 20 (PBST) and incubated with anti-mouse Alexa 555 conjugated (1:1000; Secondary antibodies, Invitrogen) or anti-rabbit IgG Alexa Fluor 488 conjugate (1:1000; Secondary antibodies, Invitrogen) for one hour, washed with PBST, and incubated with DAPI (1:1000; Sigma) for 10 min. The immunolabeled cells were examined using the Nikon Eclipse Ti-E confocal system (Nikon Instruments Inc).

In vitro angiogenesis and binding assay

For tube formation assay, human umbilical vein endothelial cells (HUVECs, $3x10^4$cells/well) were co-seeded with H9-EGFP-derived cells (MSCs, SMCs or PCs, $1.5x10^4$cells/well) or PKH67 (Sigma)-labeled brain PC (ScienCell Research) on pre-solidified MatrigelTM (BD Bioscience) in EGM-2 media (Promocell) containing 20 ng/ml VEGF. The cells were incubated for different time periods at 37^0C, 5% CO_2 in a humidified atmosphere. Vascular network was photographed at indicated time points using a Nikon Eclipse Ti-E configured with an A1R confocal system (Nikon Instruments Inc. Melville, NY) and quantified using

Wimasis tube analysis software (Wimasis Gmbh, Germany). Confocal images were also reconstructed to produce a volumetric image in 3D (three dimensional projection of the Z-stack) and converted to quick-time movies (.mov).

Gel contraction assay
Cell contractility was assessed by the gel lattice contraction assay(Dallot et al., 2003). Briefly, 8 volumes of type I collagen solution (3 mg/ml BD Bioscience) was mixed with 1 volume of 10x DMEM and 1 volume of 0.1 N NaOH on ice to yield 2 mg/ml of collagen solution at pH 7.4. A cell suspension was then made in the collagen solution on ice ($5x10^5$ cells/ml) and incubated for 2 h at 37°C for gelling, followed by the addition of medium over the gel. After allowing the cells to spread within the gel overnight, the gels were gently detached, lifted from the bottom of the well and photographed. The area of the gel lattices was determined with ImageJ software (NIMH, Bethesda, MD), and the relative lattice area was obtained by dividing the area at 48 hours of culture by the initial area of the lattice and graphed.

Time-lapse imaging for assessment of contraction and tube formation
Carbachol (Cbchl; 1mM) was added to a monolayer of MSCs, PCs or SMCs derived from H9-EGFP. Time lapse images were recorded for 15 minutes following Cbchl addition using the Nikon Eclipse Ti-E configured with an A1R confocal system, motorized stage (Nikon Instruments Inc.), and Tokai-Hit Stage Top Incubator (Tokai Hit CO., Ltd.) at 37^0C and 5% CO_2. Images were acquired continuously using Nikon Elements (NIS – element C) imaging software with CFI Plan Fluor DLL 20X NA 0.5 WD 2.1MM objective (Nikon Instruments Inc.). The time-lapse serial images were converted to Quick-time movies (.mov). Quantification of the percentage of contractile cells was measured by using ImageJ software (NIH). The percentage of contracting cells was determined from 5 different optical fields. Similarly, time-lapse imaging was also performed for the tube formation assay where HUVECs were co-seeded with H9-EGFP-derived PCs or SMCs on pre-solidified Matrigel (BD Bioscience) in EGM-2 media (Promocell) and images were acquired at 10 min intervals for 30 hrs.

Matrigel-fibrin matrix implants
In vivo vessel-stabilizing potential of MB-derived mural cells was evaluated using the implantation assay (Alajati et al., 2008) with some modifications. HUVECs were mixed with H9-EGFP ESC-derived MSCs, PCs or SMCs (2:1) in 500 µl Matrigel (growth factor reduced; BD Biosciences) and fibrinogen (final concentration of 2 mg/ml; Calbiochem) containing different growth factors (250 ng/ml each of VEGF and bFGF). Following the addition of Thrombin (0.4 U; Calbiochem) to the mixture, the matrices were injected subcutaneously on each side lateral to the abdominal midline region of 8-10 week old NOD-SCID mice. 2 weeks later, mice were sacrificed to retrieve implants. The implants were fixed overnight in 10% neutral buffered formalin, embedded in paraffin, sectioned and stained with anti-human CD31 and anti GFP antibodies (see supplementary Table 2). Blood vessels and recruitment of mural cells to vessels were counted in 10 microscopic fields and averages were taken. All animal procedures were performed under protocols approved by University of Wisconsin Institutional Animal Care and Use Committee.

Quantitative Real-Time PCR
Total RNA was extracted using the RNeasy mini Kit (Qiagen) according to the manufacturers's instructions. RNA yield was determined by using the NanoDrop ND-1000 spectrophotometer (NanoDrop Technologies). Total RNA (0.5µg) was converted to cDNA

using Advantage RT-for-PCR Kit (Clontech). Quantitative real-time PCR analysis was performed for all the cDNA samples using self-designed specific primers (Table S3) and Power SYBR Green PCR master mix (Life Technologies). The reactions were run on a Mastercycler realplex thermal cycler (Eppendorf) and expression levels were calculated by minimal cycle threshold values (Ct) normalized to the reference expression of GAPDH in each sample (Pfaffl, 2001).

Low level RNA-Seq data processing

Total RNA was isolated from the in vitro generated cell subsets, somatic placental (passage 4; PromoCell), retinal PCs (passage 4; Cell System) and aortic SMCs (passage 3, Lonza) using the RNeasy mini Kit (Qiagen). DNaseI treatment was performed in the column according to the manufacture's protocol. Total mRNA from brain PCs was purchased from ScienCell Research Laboratories. RNA purity and integrity was evaluated by capillary electrophoresis on the Bioanalyzer 2100 (Agilent Technologies, Santa Clara, CA). Samples were then prepared for sequencing using the Ligation Mediated Sequencing (LM-Seq) protocol, according to the published guidelines (Hou et al., 2015). Final sample libraries were quantitated with the Life Technologies Qubit fluorometer and sequenced on the Illumina HiSeq 2500 (SY-401-1003-PRE). Base-calling and demultiplexing were completed with the Illumina Genome Analyzer Casava Software, version 1.8.2. Following quality assessment and filtering for adapter molecules and other sequencing artifacts, the remaining sequencing reads were aligned to 19084 RefSeq genes extracted from the Illumina iGenomes annotation, selecting only "NM_" designated genes. Bowtie v 0.12.9 was used, allowing two mismatches in a 28 bp seed, and excluding reads with more than 200 alignments (Langmead et al., 2009). RSEM v 1.2.3 was used to estimate isoform or gene relative expression levels in units of ''transcripts per million'' (tpm), as well as "expected counts" (the non-normalized absolute number of reads assigned by RSEM to each isoform/gene) (Li and Dewey, 2011; Li et al., 2010). R statistical environment (R core team, 2014) was used at all of the stages of downstream data analysis. The entire set of libraries was pre-normalized as a pool using median normalization routine from EBSeq package (Leng et al., 2013).

Downstream RNAseq bioinformatics analysis

Testing for differential expression

The entire set of libraries was pre-normalized as a pool using median normalization routine from EBSeq package (Leng et al., 2013). EBSeq with 10 iterations was applied to call for differential expression. The EBSeq's default procedure of filtering low-expressed genes was suppressed by setting the *QtrmCut* parameter to zero. Genes with assigned value of Posterior Probability of Differential Expression above 0.95 were preliminary selected. Subsequently, only genes demonstrating the Critical Coefficent (Moskvin et al., 2014) value above 1.2 were retained as differentially expressed. The latter also addressed the higher noise issue of low-expressed genes while preserving sensitivity for differential expression detection.

Identification of cell type-specific expression markers

Efficient detection of expression markers required modifications to the gene selection routine due to the fact that a consistent fold change cutoff results in high bias towards low-expressed genes which are less attractive candidates for followup. To retain the most interesting of low-expressed genes showing extremely high expression changes, in addition to calling highly expressed marker genes that may not demonstrate an extreme expression change, we introduced a hyperbolic-exponential filtering procedure that represents an

adaptive expression change filter that depends on the expression value in the target cell type. As a measure of expression change, the critical coefficient rather than conventional fold change was applied; this allowed for cleaner separation of marker and non-marker genes (not shown). Genes pre-selected by the differential expression test routine (FDR < 0.05 and critical coefficient 2, upregulation in the target cell type) were called cell type markers if they additionally satisfied the following empirical condition:
$log10(crt) >= 0.6/(log10(TPM)-0.74)^{4.2} + 0.225$ where crt is the critical coefficient, TPM is Transcripts Per Million measure in the cell population of interest. Graphical justification of the filter is presented in Figure S7. The R script and detailed instructions on application of the hyperbolic-exponential filter, including more parameters for added flexibility are available at https://github.com/scienceforever/HypExpo . To generate a filter corresponding to the above equation, parameters of the HypExpo function were set to the following values: param.hyp=0.6, param.expo=4.2, param.diag=0, param.shift=0.225, param.xshift=0.74. This resulted in sufficiency of the minimal fold change 2 to call markers all the genes with expression level in the target cells above 237 TPM, with gradual increase of the required cutoff to 3 for expression level of 93 TPM, 4 to expression level 72 TPM, 10 for expression level 47 TPM etc., rising the required minimal fold change to prohibitively high values (in the range of millions) when the expression goes down to 20 or lower.

Gene set enrichment analysis
Functionality enrichment tests with lists of responsive genes were performed with Fisher's genome-wide summarization of p-values in context of gene sets, using Bioconductor *piano* package (Varemo et al., 2013) that was supplied with custom reference gene sets. KEGG gene-pathway assignments were downloaded via Bioconductor's KEGGREST (Tenenbaum, 2013) package. GeneOntology assignments for *Homo sapience* genes were adopted from GSEA website of Broad Institute (Subramanian et al., 2005). Statistical significance of the enrichment tests was estimated with 100,000 data permutations.

Principal Component Analysis
PCA was performed with TPM (transcripts per million) normalized data using "stats" package for R programming language, with "scale" option enabled. The first three principal components described 46.7% of the data variation. RankShift transformation was applied to the TPM values prior to PCA. The detailed justification and benchmarking of the RankShift procedure will be published elsewhere. Briefly, genes were ranked by their expression values within each library, and after that, the expression values were replaced with the difference between their within-library rank and the median of within-library ranks across all the samples. The transformation significantly improved the separation of cell types and clustering accuracy of the biological replicates at the same time.

Statistical Analysis

Statistical analysis was performed using GraphPad Prism Software (GraphPad). Data obtained from multiple experiments were reported as the mean $\pm$ SE. The significance of difference between the mean values was determined by paired Student's t test. Differences were considered significant when $p<0.01$.

Supplementary References

Alajati, A., Laib, A.M., Weber, H., Boos, A.M., Bartol, A., Ikenberg, K., Korff, T., Zentgraf, H., Obodozie, C., Graeser, R., *et al.* (2008). Spheroid-based engineering of a human vasculature in mice. Nat Methods *5*, 439-445.
Dallot, E., Pouchelet, M., Gouhier, N., Cabrol, D., Ferre, F., and Breuiller-Fouche, M. (2003). Contraction of cultured human uterine smooth muscle cells after stimulation with endothelin-1. Biol Reprod *68*, 937-942.
Hou, Z., Jiang, P., Swanson, S.A., Elwell, A.L., Nguyen, B.K., Bolin, J.M., Stewart, R., and Thomson, J.A. (2015). A cost-effective RNA sequencing protocol for large-scale gene expression studies. Sci Rep *5*, 9570.
Hu, Y., and Smyth, G.K. (2009). ELDA: extreme limiting dilution analysis for comparing depleted and enriched populations in stem cell and other assays. J Immunol Methods *347*, 70-78.
Langmead, B., Trapnell, C., Pop, M., and Salzberg, S.L. (2009). Ultrafast and memory-efficient alignment of short DNA sequences to the human genome. Genome Biol *10*, R25.
Leng, N., Dawson, J.A., Thomson, J.A., Ruotti, V., Rissman, A.I., Smits, B.M., Haag, J.D., Gould, M.N., Stewart, R.M., and Kendziorski, C. (2013). EBSeq: an empirical Bayes hierarchical model for inference in RNA-seq experiments. Bioinformatics *29*, 1035-1043.
Lex, A., Gehlenborg, N., Strobelt, H., Vuillemot, R., and Pfister, H. (2014). UpSet: Visualization of Intersecting Sets. Ieee T Vis Comput Gr *20*, 1983-1992.
Li, B., and Dewey, C.N. (2011). RSEM: accurate transcript quantification from RNA-Seq data with or without a reference genome. BMC Bioinformatics *12*, 323.
Li, B., Ruotti, V., Stewart, R.M., Thomson, J.A., and Dewey, C.N. (2010). RNA-Seq gene expression estimation with read mapping uncertainty. Bioinformatics *26*, 493-500.
Moskvin, O.V., McIlwain, S., and Ong, I.M. (2014). CAMDA 2014: Making sense of RNA-Seq data: from low-level processing to functional analysis. . Systems Biomedicine *2*, 31-40.
Pfaffl, M.W. (2001). A new mathematical model for relative quantification in real-time RT-PCR. Nucleic Acids Res *29*, e45.
Subramanian, A., Tamayo, P., Mootha, V.K., Mukherjee, S., Ebert, B.L., Gillette, M.A., Paulovich, A., Pomeroy, S.L., Golub, T.R., Lander, E.S., *et al.* (2005). Gene set enrichment analysis: a knowledge-based approach for interpreting genome-wide expression profiles. Proceedings of the National Academy of Sciences of the United States of America *102*, 15545-15550.
Varemo, L., Nielsen, J., and Nookaew, I. (2013). Enriching the gene set analysis of genome-wide data by incorporating directionality of gene expression and combining statistical hypotheses and methods. Nucleic Acids Res *41*, 4378-4391.
Vodyanik, M.A., and Slukvin, II (2007). Hematoendothelial differentiation of human embryonic stem cells. Curr Protoc Cell Biol *Chapter 23*, Unit 23 26.
Vodyanik, M.A., Yu, J., Zhang, X., Tian, S., Stewart, R., Thomson, J.A., and Slukvin, II (2010). A mesoderm-derived precursor for mesenchymal stem and endothelial cells. Cell Stem Cell *7*, 718-729.
